# Oncogenic miR-210-3p promotes prostate cancer cell EMT and bone metastasis via NF-κB signaling pathway

**DOI:** 10.1186/s12943-017-0688-6

**Published:** 2017-07-10

**Authors:** Dong Ren, Qing Yang, Yuhu Dai, Wei Guo, Hong Du, Libing Song, Xinsheng Peng

**Affiliations:** 1 Department of Orthopaedic Surgery, the First Affiliated Hospital of Sun Yat-sen University, 58# Zhongshan 2rd Road, Guangzhou, 510080 China; 2 Guangdong Provincial Key Laboratory of Orthopedics and Traumatology, Guangdong Province, Guangzhou, 510080 China; 3 Department of Pathology, the First People’s Hospital of Guangzhou City, Guangdong Province, Guangzhou, 510180 China; 4 Department of Experimental Research, State Key Laboratory of Oncology in Southern China, Sun Yat-sen University Cancer Center, Guangzhou, 510060 China

**Keywords:** miR-210-3p, EMT, Bone metastasis, NF-κB signaling and prostate cancer

## Abstract

**Background:**

The primary issue arising from prostate cancer (PCa) is its high prevalence to metastasize to bone, which severely affects the quality of life and survival time of PCa patients. miR-210-3p is a well-documented oncogenic miRNA implicated in various aspects of cancer development, progression and metastasis. However, the clinical significance and biological roles of miR-210-3p in PCa bone metastasis remain obscure.

**Methods:**

miR-210-3p expression was evaluated by real-time PCR in 68 bone metastatic and 81 non-bone metastatic PCa tissues. The biological roles of miR-210-3p in the bone metastasis of PCa were investigated both in vitro by EMT and Transwell assays, and in vivo using a mouse model of left cardiac ventricle inoculation. Bioinformatics analysis, real-time PCR, western blot and luciferase reporter analysis were applied to discern and examine the relationship between miR-210-3p and its potential targets. RT-PCR was performed to identify the underlying mechanism of miR-210-3p overexpression in bone metastasis of PCa. Clinical correlation of miR-210-3p with its targets was examined in human PCa and metastatic bone tissues.

**Results:**

miR-210-3p expression is elevated in bone metastatic PCa tissues compared with non-bone metastatic PCa tissues. Overexpression of miR-210-3p positively correlates with serum PSA levels, Gleason grade and bone metastasis status in PCa patients. Upregulating miR-210-3p enhances, while silencing miR-210-3p represses the EMT, invasion and migration of PCa cells in vitro. Importantly, silencing miR-210-3p significantly inhibits bone metastasis of PC-3 cells in vivo. Our results further demonstrate that miR-210-3p maintains the sustained activation of NF-κB signaling via targeting negative regulators of NF-κB signaling (TNF-α Induced Protein 3 Interacting Protein 1) TNIP1 and (Suppressor Of Cytokine Signaling 1) SOCS1, resulting in EMT, invasion, migration and bone metastasis of PCa cells. Moreover, our results further indicate that recurrent gains (amplification) contribute to miR-210-3p overexpression in a small number of PCa patients. The clinical correlation of miR-210-3p with SOCS1, TNIP1 and NF-κB signaling activity is verified in PCa tissues.

**Conclusion:**

Our findings unravel a novel mechanism for constitutive activation of NF-κB signaling pathway in the bone metastasis of PCa, supporting a functional and clinical significance of epigenetic events in bone metastasis of PCa.

**Electronic supplementary material:**

The online version of this article (doi:10.1186/s12943-017-0688-6) contains supplementary material, which is available to authorized users.

## Background

Prostate cancer (PCa) is the most common malignant cancer and the second leading cause of cancer-related death in men worldwide [1]. The primary issue derived from PCa is its propensity to metastasize to bone, which occurred in up to 90% of patients with advanced PCa [2]. Despite great advances in systemic and individualized treatments of PCa in the last decades, distant bone metastasis remains a principal issue, which severely affects the quality of life and survival time of PCa patients [3]. Thus, it is of great importance to better understand the underlying mechanisms contributing to bone metastasis of PCa, which will facilitate the development of novel anti-bone metastasis therapeutic avenues in PCa.

Epithelial-mesenchymal transition (EMT) is an imperative phenotypic conversion that occurs during several processes, including embryonic development, tissue remodelling and metastasis, where epithelial cells obtain mesenchymal-like properties in combination with reduced intercellular adhesion and enhanced motility [4, 5]. EMT is a transient and dynamic process that primarily emerges at the onset of invasion and is tightly controlled by several cellular signaling pathways, such as ErbB, Wnt, NF-kB and TGF-β pathways [6–8]. Among these, transforming growth factor (TGF)-β is identified as the most important inducer of EMT process due to stimulation of the expression of EMT-inducing transcription factors, including Snail1, Twist1 and ZEB1/2 [9–11]. Furthermore, accumulating studies have demonstrated that NF-kB signaling pathway is essential for the induction and maintenance of EMT in a large number of cancers [7, 12, 13]. The NF-κB pathway was discovered nearly three decades ago [14], and the critical roles of the NF-κB pathway in physiologic processes, such as immunity and inflammation, have been well documented [15, 16]. NF-κB signaling has been reported to be constitutively activated in a number of human cancers, which contributed to the initiation and progression of a large array of malignancies [15, 17]. Furthermore, accumulating literatures reported that NF-κB signaling plays a crucial role in the bone metastasis of various types of cancers [18, 19]. Park and colleagues reported that constitutive NF-κB activity in breast cancer cells was crucial for the bone resorption characteristic of the osteolytic bone metastasis via transcriptionally regulating granulocyte macrophage-colony stimulating factor (GM-CSF) that mediated osteolytic bone metastasis of breast cancer by stimulating osteoclast development [20]. Furthermore, several lines of evidence have implied that NF-κB activation was also associated with the metastatic phenotype of PCa progression [19, 21]. Chen et al. reported that NF-κB activation was crucial for the development of PCa bone metastasis [19]. However, the underlying mechanism responsible for constitutive activation of NF-κB signaling in the bone metastasis of PCa remains largely unknown.

MicroRNAs (miRNAs) are small endogenous non-coding RNAs that are responsible for post-transcriptional regulation of target genes by binding with specific sequences in the 3′ untranslated region (3’UTR) of downstream target genes, leading to mRNA degradation and/or translational inhibition [22]. miRNAs play important roles in many cellular and biological processes such as proliferation, apoptosis, differentiation, metabolism, cardiogenesis, development and function of the nervous and immune systems [22, 23]. The dysregulation of miRNAs in cancers is widely documented, and several studies have revealed a correlation of miRNAs expression levels and metastatic tumors [24, 25]. Furthermore, several miRNAs have been reported as mediators in the bone metastasis of PCa [26, 27]. Our previous studies demonstrated that loss of wild-type P53 induced downregulation of miR-145 promoted bone metastasis of PCa via regulating several positive regulators of EMT [28–30]. These studies indicate that aberrant expression of miRNAs elicited by unknown mechanism plays a crucial role in the bone metastasis of PCa.

In this study, we report that miR-210-3p expression is elevated in PCa tissues compared with the adjacent prostate epithelial tissues (ANT). Interestingly, the expression levels of miR-210-3p increases steadily from non-bone metastatic PCa tissues, bone metastatic PCa tissues to metastatic bone tissues and high expression of miR-210-3p positively correlates with the clinicopathological characteristics and bone metastasis status of PCa patients. Furthermore, upregulating miR-210-3p enhances, while silencing miR-210-3p suppresses the EMT, invasion and migration of PCa cells in vitro. Importantly, silencing miR-210-3p significantly inhibits bone metastasis of PC-3 cells in vivo. Furthermore, our results demonstrate that miR-210-3p promotes EMT, invasion and migration of PCa cells via targeting negative regulators of NF-κB signaling (TNF-α Induced Protein 3 Interacting Protein 1) TNIP1 and (Suppressor Of Cytokine Signaling 1) SOCS1, resulting in constitutive activation of NF-κB signaling pathway. Our results further indicate that recurrent gains are responsible for miR-210-3p overexpression in a small number of PCa patients. The analysis of clinical correlation reveals that miR-210-3p inversely correlates with SOCS1 and TNIP1, but positively correlates with NF-κB signaling activity in human PCa and metastatic bone tissues. Taken together, these findings uncover a plausible mechanism responsible for constitutive activation of NF-κB signaling in bone metastasis of PCa, suggesting that miR-210-3p may serve as a novel target for clinical intervention in PCa.

## Methods

### Cell culture and hypoxic condition

The human PCa cell lines 22RV1, PC-3, VCaP, DU145, LNCaP and normal prostate epithelial cells RWPE-1 were obtained from Shanghai Chinese Academy of Sciences cell bank (China). RWPE-1 cells were grown in defined keratinocyte-SFM (1×) (Invitrogen). PC-3, LNCaP and 22Rv1 cells were cultured in RPMI-1640 medium (Life Technologies, Carlsbad, CA, US) supplemented with penicillin G (100 U/ml), streptomycin (100 mg/ml) and 10% fetal bovine serum (FBS, Life Technologies). DU145 and VCaP cells were grown in Dulbecco’s modified Eagle’s medium (Invitrogen) supplemented with 10% FBS. The C4-2B cell line was purchased from the MD Anderson Cancer Center and maintained in T-medium (Invitrogen) supplemented with 10% FBS [31]. All cell lines were grown under a humidified atmosphere of 5% CO2 at 37 °C. A hypoxic condition was induced via culturing the cells under 1% oxygen tension (1% O2) in a hypoxia chamber for 24–48 h, as previously described [32], as well as treated the cells with 50–200 μmol L^−1^ cobalt chloride (CoCl2) for 24 h to mimic the hypoxic condition by stabilization of HIF-1a [33].

### Plasmid, small interfering RNA and transfection

The human miR-210-3p gene was PCR-amplified from genomic DNA and cloned into a pMSCV-puro retroviral vector (Clontech, Japan). The pNFκB-luc and control plasmids (Clontech, Japan) were used to examine the activity of transcription factor quantitatively. The 3′-untranslated region (3’UTR) regions of the human SOCS1 and TNIP1 were PCR-amplified from genomic DNA and cloned into pmirGLO vectors (Promega, USA), and the list of primers used in cloning reactions is presented in Additional file 1: Table S1. Antagomir-210-3p, small interfering RNA (siRNA) for the SOCS1 and TNIP1 knockdown and conresponding control siRNAs were synthesized and purified by RiboBio. Transfection of miRNA, siRNAs, and plasmids was performed using Lipofectamine 3000 (Life Technologies, USA) according to the manufacturer’s instructions.

### RNA extraction, reverse transcription, and real-time PCR

Total RNA from tissues or cells was extracted using the RNA Isolation Kit (Qiagen, USA) according to the manufacturer’s instructions. Messenger RNA (mRNA) and miRNA were reverse transcribed from total mRNA using the RevertAid First Strand cDNA Synthesis Kit (Thermo Fisher, USA) according to the manufacturer’s protocol. Complementary DNA (cDNA) was amplified and quantified on the CFX96 system (BIO-RAD, USA) using iQ SYBR Green (BIO-RAD, USA). The primers are provided in Additional file 2: Table S2. The analysis procedure of amplification level in PCa tissues was as following: examine the CNV of each sample of prostate cancer using Real time PCR primer Hs03772990_cn through TaqMan Copy Number Assay; TaqMan Copy Number Reference Assay RNase P and TaqMan Fast Advanced Master Mix were used as the loading control and amplification kit; procure the CNV number of each corresponding sample and define the CNV number of Amplification and Gain groups as “Gain” and the rest as “No Gain”; analyze the result using Excel 2010 and depict each figure respectively by GraphPad 5 software. Primers for U6 and miR-210-3p were synthesized and purified by RiboBio (Guangzhou, China). U6 or glyceraldehyde-3-phosphate dehydrogenase (GAPDH) was used as the endogenous controls. Relative fold expressions were calculated with the comparative threshold cycle (2^-ΔΔCt^) method.

### Patients and tumor tissues

A total of 149 archived PCa tissues, including 81 non-bone metastatic PCa tissues and 68 bone metastatic PCa tissues, and 9 metastatic bone tissues were obtained during surgery or needle biopsy at The First People’s Hospital of Guangzhou City (Guangzhou, China) between January 2008 and October 2016. Patients were diagnosed based on clinical and pathological evidence, and the specimens were immediately snap-frozen and stored in liquid nitrogen tanks. For the use of these clinical materials for research purposes, prior patient’ consents and approval from the Institutional Research Ethics Committee were obtained. The clinicopathological features of the patients are summarized in Additional file 3: Table S3. The median of miR-210-3p expression in PCa tissues was used to stratify high and low expression of miR-210-3p.

### High throughput data processing

Copy number variation profile of prostate cancer dataset was downloaded from The Cancer Genome Atlas (TCGA; https://gdc.cancer.gov/). The analysis method for copy number variation profile was as following: download the Level 3 Copy Number Variation (CNV) dataset of prostate cancer in SNP6.0 microarray from TCGA; analyze the dataset by GISTIC2.0 software as described previously (all parameters as the default) [34]; procure the CNV number of each corresponding sample and define the CNV number of Amplification and Gain groups as “Gain” and the rest as “No Gain”; analyze the result using Excel 2010 and depict each figure using GraphPad 5 software.

### Western blotting

Nuclear/cytoplasmic fractionation was separated using the Cell Fractionation Kit (Cell Signaling Technology, USA) according to the manufacturer’s instructions, and the whole cell lysates were extracted with RIPA Buffer (Cell Signaling Technology). Western blotting was performed according to a standard method, as described previously [35]. Antibodies against E-cadherin (Cat# 3195), Vimentin (Cat# 5741), Fibronectin (Cat# 4706), SOCS1 (Cat# 3950), TNIP1 (Cat# 4664) and PIAS4 (Cat# 4392) were purchased from Cell Signaling Technology, and p65 (cat# 10745–1-AP) from Proteintech, p84 (Cat#:PA5–27816) from Invitrogen and PDLIM7 (Cat#:SAB1406807) from Sigma-Aldrich,USA. The membranes were stripped and reprobed with an anti–α-tubulin antibody (Sigma-Aldrich, USA) as the loading control.

### Luciferase assay

Cells (4 × 10^4^) were seeded in triplicate in 24-well plates and cultured for 24 h. Cells were transfected with 100 ng of the pNFκB reporter luciferase plasmid, or pmirGLO-SOCS1–3′UTR, or –TNIP1–3′UTR luciferase plasmid, plus 5 ng pRL-TK the Renilla plasmid (Promega) using Lipofectamine 3000 (Invitrogen) according to the manufacturer’s recommendations. Luciferase and Renilla signals were measured 36 h after transfection using a Dual Luciferase Reporter Assay Kit (Promega) according to the manufacturer’s protocol.

### miRNA immunoprecipitation

Cells were co-transfected with HA-Ago2, followed by HA-Ago2 immunoprecipitation using anti-HA-antibody. Real-time PCR analysis of the IP material was performed to test the association of the mRNA of SOCS1 and TNIP1 with the RISC complex. The specific processes were performed as previously described [36].

### Invasion and migration assays

The invasion and migration assays were performed using Transwell chamber consisting of 8-mm membrane filter inserts (Corning) with or without coated Matrigel (BD Biosciences) respectively as described previously [37]. Briefly, the cells were trypsinized and suspended in serum-free medium. Then, 1.5 × 10^5^ cells were added to the upper chamber, and lower chamber was filled with the culture medium supplemented with 10% FBS. After incubation for 24–48 h, cells passed through the coated membrane to the lower surface, where cells were fixed with 4% paraformaldehyde and stained with haematoxylin. The cell count was performed under a microscope (×100).

### Animal study

All mouse experiments were approved by The Institutional Animal Care and Use Committee of Sun Yat-sen University and the approval-No. was L102012016110D. For the bone metastasis study, BALB/c-nu mice ((5–6 weeks old, 18–20 g)) were anaesthetized and inoculated into the left cardiac ventricle with 1 × 10^5^ PC-3 cells in 100 μl of PBS. Bone metastases were monitored by bioluminescent imaging (BLI) as previously described [38]. Osteolytic lesions were identified on radiographs as radiolucent lesions in the bone. The area of the osteolytic lesions was measured using the Metamorph image analysis system and software (Universal Imaging Corporation), and the total extent of bone destruction per animal was expressed in square millimeters. Each bone metastasis was scored based on the following criteria: 0, no metastasis; 1, bone lesion covering <1/4 of the bone width; 2, bone lesion involving 1/4 ~ 1/2 of the bone width; 3, bone lesion across 1/2 ~ 3/4 of the bone width; and 4, bone lesion >3/4 of the bone width. The bone metastasis score for each mouse was the sum of the scores of all bone lesions from four limbs. For survival studies, mice were monitored daily for signs of discomfort, and were either euthanized all at one time or individually when presenting signs of distress, such as a 10% loss of body weight, paralysis, or head tilting.

### Statistical analysis

All values are presented as the mean ± standard deviation (SD). Significant differences were determined using the GraphPad 5.0 software (USA). Student’s t-test was used to determine statistical differences between two groups. The chi-square test was used to analyze the relationship between miR-210-3p expression and clinicopathological characteristics. *P* < 0.05 was considered significant. All experiments were repeated three times.

## Results

### miR-210-3p expression is elevated in PCa, particularly in bone-metastatic PCa

**Fig. 1:**
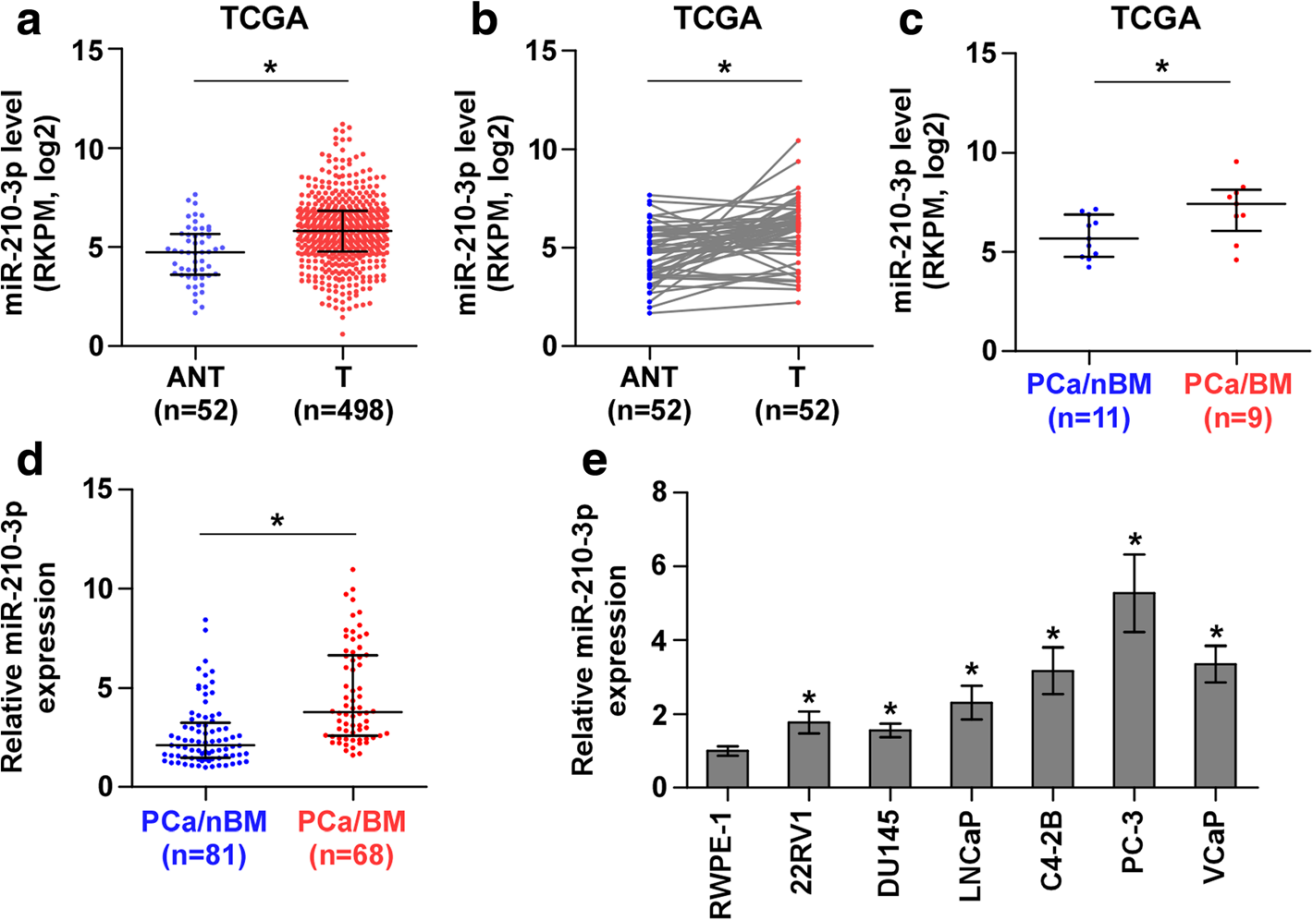
miR-210-3p is upregulated in bone metastatic PCa tissues and cells. **a** miR-210-3p expression levels was markedly elevated in PCa tissues compared with that in adjacent normal tissues (ANT) as assessed by analyzing the TCGA PCa miRNA sequencing dataset (ANT, *n* = 52; PCa, *n* = 498). *P < 0.05. **b** Real-time PCR analysis of miR-210-3p in 52 paired PCa tissues compared with the matched adjacent normal tissues as assessed by analyzing the TCGA PCa miRNA sequencing dataset. *P < 0.05. **c** miR-210-3p expression levels was markedly elevated in bone metastatic PCa tissues (PCa/BM) compared with that in non-bone metastatic PCa tissues (PCa/nBM) as assessed by analyzing the TCGA PCa miRNA sequencing dataset (PCa/nBM, *n* = 11; PCa/BM, *n* = 9).*P< 0.05. **d** Real-time PCR analysis of miR-210-3p expression in 81 non-bone metastatic and 68 bone metastatic PCa samples. Transcript levels were normalized to *U6* expression. Lines represent median and lower/upper quartiles. *P < 0.05. **e** Real-time PCR analysis of miR-210-3p expression levels in normal prostate epithelial cell (RWPE-1), primary PCa cell 22RV1, bone metastatic PCa cell lines (PC-3, C4-2B and VCaP) and brain metastatic cell line DU145 and lymph node metastatic cell line LNCaP. Transcript levels were normalized to *U6* expression. *P < 0.05

To determine the clinical significance of miR-210-3p in PCa, we first analyzed the miRNA sequencing dataset of PCa from The Cancer Genome Atlas (TCGA) and found that miR-210-3p expression was elevated in PCa tissues compared with the adjacent normal tissues (ANT) (Fig. 1a and b). Interestingly, miR-210-3p expression was further higher in bone metastatic PCa tissues than in non-bone metastatic PCa tissues (Fig. 1c). We further examined the expression levels of miR-210-3p in our 149 PCa tissues and found that the miR-210-3p expression level in bone metastatic PCa tissues was robustly elevated compared with non-bone metastatic PCa tissues (Fig. 1d). Furthermore, the percentage of high expression of miR-210-3p was higher in bone metastatic PCa tissues than in non-bone metastatic PCa tissues (Additional file 4: Fig. S1a). Consistent with the miR-210-3p expression in PCa tissues, miR-210-3p expression was elevated in PCa cells compared with normal prostate epithelial cells RWPE-1 (Fig. 1e and Additional file 4: Figure S1b). Importantly, the miR-210-3p expression levels in bone metastatic PCa cell lines (PC-3, C4-2B and VCaP) were differentially higher than in primary PCa cell 22RV1 and brain metastatic cell line DU145 and lymph node metastatic cell line LNCaP (Fig. 1e). Statistical analysis of PCa tissue samples revealed that miR-210-3p overexpression strongly correlated with serum PSA levels, Gleason grade and bone metastasis status in PCa (Additional file 3: Table S3 and Additional file 5: Table S4). Collectively, these results indicate that overexpression of miR-210-3p is involved the bone metastasis of PCa.

### Silencing miR-210-3p inhibits bone metastasis of PC-3 cells in vivo

**Fig. 2:**
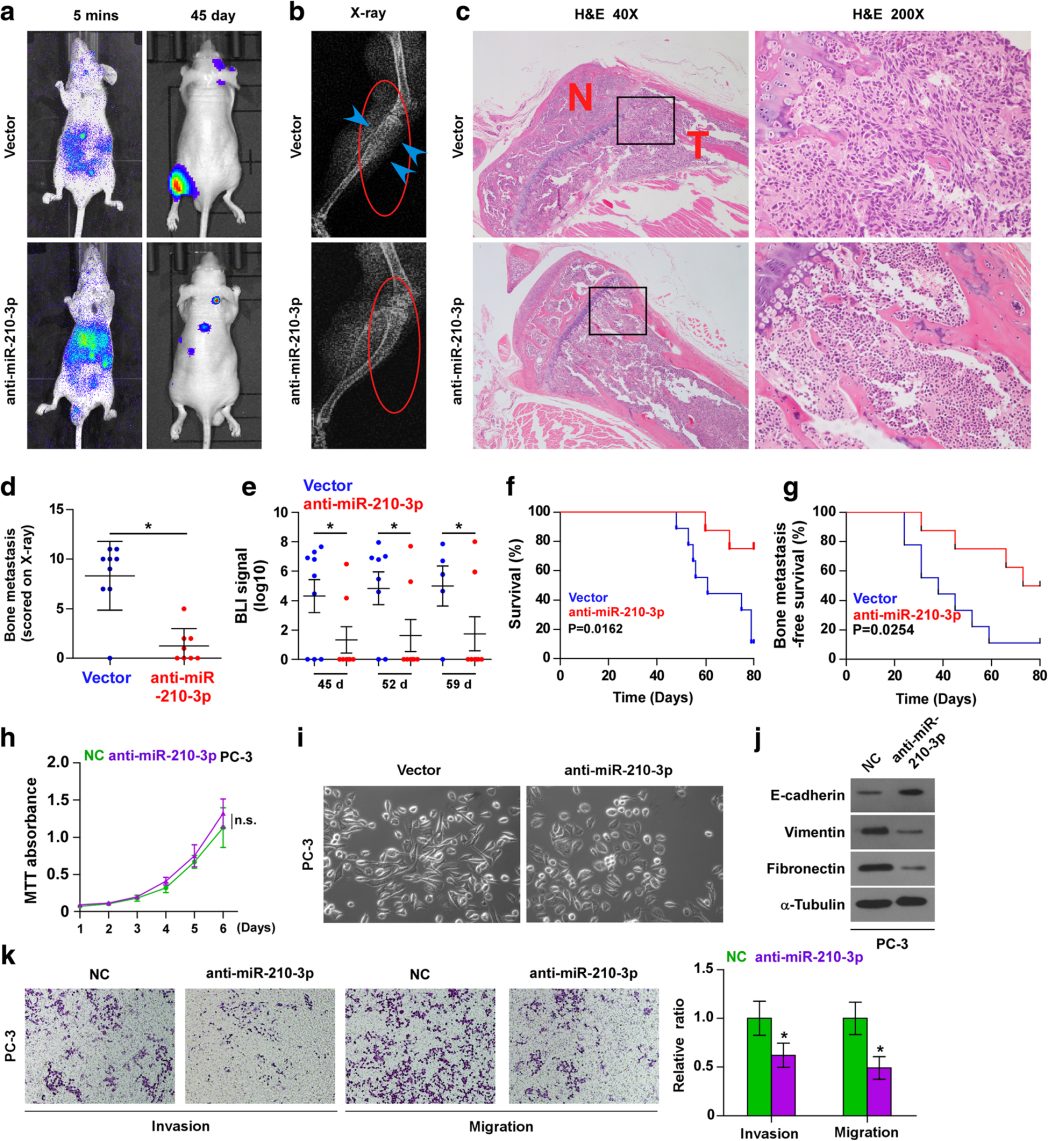
Silencing miR-210-3p inhibits bone metastasis of PC-3 cells in vivo. **a** Representative BLIs signal of bone metastasis of a mouse from the indicated groups of mice at 5 mins and 45 day respectively. **b** Representative radiographic images of bone metastases in the indicated mice (arrows indicate osteolytic lesions). **c** Representative H&E-stained sections of tibias from the indicated mouse (T, tumor; N, the adjacent non-tumor tissues). **d** The sum of bone metastasis scores for each mouse in tumor-bearing mice inoculated with vector (*n* = 9) or anti-miR-210-3p (*n* = 8) cells.​ *P < 0.05. **e** Quantification of the BLI signaling in the vector and miR-210-3p-downexpression groups at 45, 52 and 59 day respectively. *P < 0.05. **f** Kaplan-Meyer analysis of mouse survival in the vector and miR-210-3p-downexpression groups. **g** Kaplan-Meier analysis of mouse bone metastasis-free survival in the vector and miR-210-3p-downexpression groups. **h** Silencing miR-210-3p had no obvious effect on proliferation ability of PC-3 cells as assessed by MTT assay of proliferation (n=3). n.s. means no significance. **i** Silencing miR-210-3p converted a stick-like or long spindleshaped mesenchymal profile to a cobblestone-like or a short spindle-shaped epithelial morphology in PC-3 cells. **j** Silencing miR-210-3p increased E-cadherin expression and decreased Vimentin and Fibronectin expression in PC-3 cells. α-Tubulin served as the loading control. **k** Silencing miR-210-3p suppressed invasion and migration abilities in PC-3 cells. Error bars represent the mean ± S.D. of three independent experiments. *P < 0.05

To determine the effect of miR-210-3p on the bone metastasis of PCa in vivo, we first endogenously silenced miR-210-3p by transfecting anti-miR-210-3p in PC-3 cells based on the expression level of miR-210-3p shown in Fig. 2e (Additional file 6: Figure S2). To establish a rapid mouse model of bone metastasis, the luciferase-labeled vector or miR-210-3p-silencing PC-3 cells were inoculated perspectively into the left cardiac ventricle of male nude mice to monitor the progression of bone metastasis by bioluminescence imaging (BLI). As shown in Fig. 2a and b, the miR-210-3p-silenced PC-3 cells displayed lower bone metastasis ability compared with the control group by X-ray and BLI. H&E staining of the tumor sections from the tibias of injected mice demonstrated that silencing miR-210-3p dramatically reduecd the tumor burden in bone (Fige. 2c).Furthermore, miR-210-3p silenced cells exhibited fewer bone metastatic sites and smaller osteolytic area of metastatic tumors, as well as longer survival and bone metastasis-free survival compared to the control group (Fig. 2e-g). The effect of silencing miR-210-3p on proliferation was not cytotoxic as assessed by MTT assay of proliferation in PC-3 cells (Fig. 2h). Consequently, these finding demonstrate that silencing miR-210-3p inhibits the bone metastasis of PCa in vivo.

### miR-210-3p promotes EMT, migration and invasion in PCa cells

**Fig. 3:**
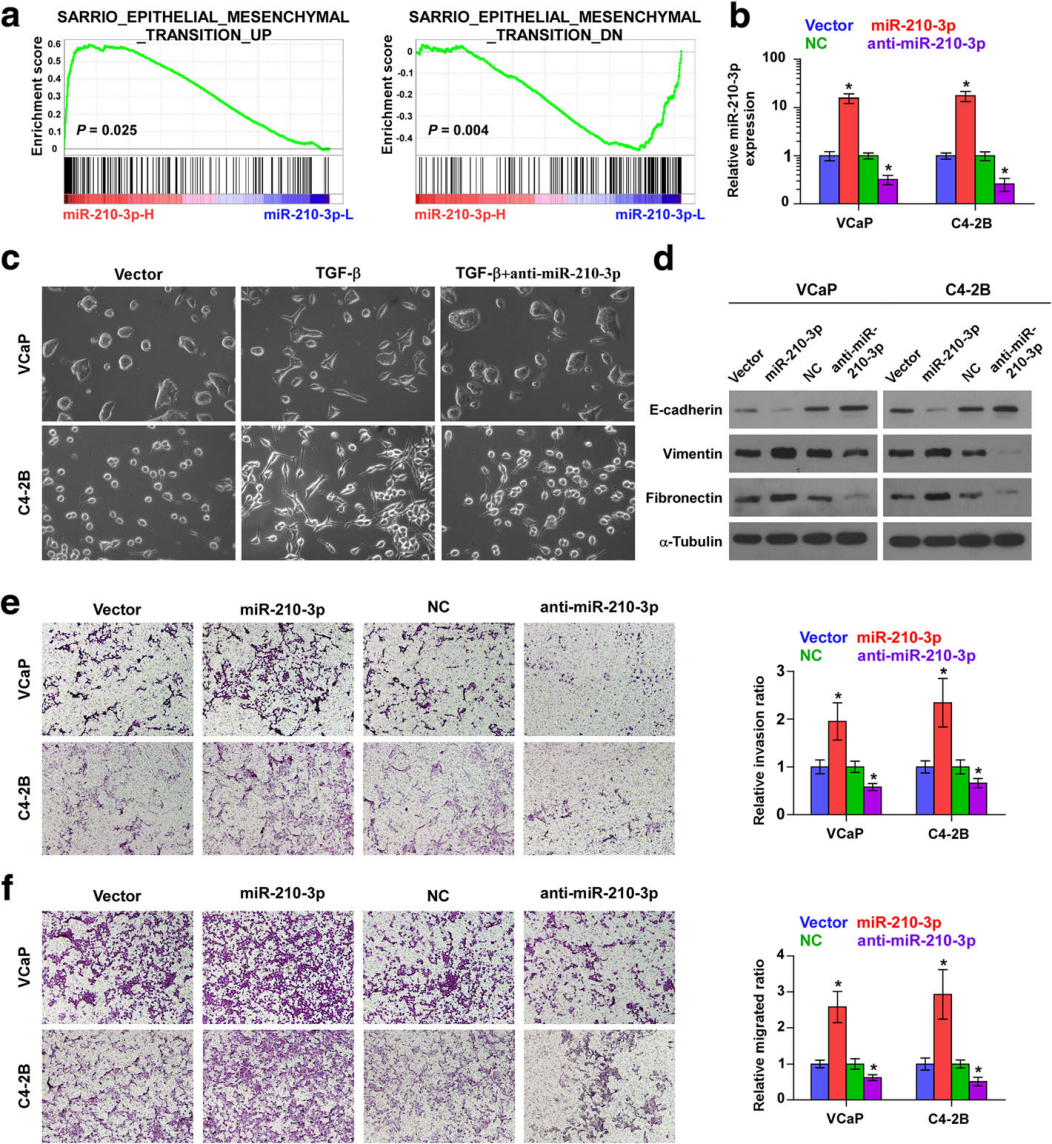
miR-210-3p promotes EMT, invasion and migration in PCa cells in vitro. **a** Gene set enrichment analysis (GSEA) revealed that miR-210-3p expression significantly and positively correlated with the EMT signatures. **b** Real-time PCR analysis of miR-210-3p expression inVCaP and C4-2B cells transduced with miR-210-3p or transfected with anti-miR-210-3p compared to controls. Transcript levels were normalized by *U6* expression. Error bars represent the mean ± s.d. of three independent experiments. *P < 0.05. **c** Silencing miR-210-3p converted a stick-like or long spindleshaped mesenchymal profile to a cobblestone-like or a short spindle-shaped epithelial morphology in VCaP and C4-2B cells treated with TGF-β (5 ng/ml for 72 h). **d** Overexpression of miR-210-3p decreased E-cadherin expression and increased Vimentin and Fibronectin expression in VCaP and C4-2B cells; while silencing miR-210-3p increased E-cadherin expression and decreased Vimentin and Fibronectin expression in VCaP and C4-2B cells. α-Tubulin served as the loading control. **e** and **f** Overexpression of miR-210-3p enhanced, while silencing miR-210-3p suppressed invasion (**e**) and migration (**f**} abilities in VCaP and C4-2B cells. Error bars represent the mean ± S.D. of three independent experiments. *P < 0.05

The biological role of miR-210-3p in bone metastasis of PCa was first analyzed by Gene Set Enrichment Analysis (GSEA) based on mRNA expression data from TCGA, and the result showed that miR-210-3p expression level positively correlated with EMT-associated gene signatures (Fig.3a). Then, we further exogenously overexpressed miR-210-3p and endogenously silenced miR-210-3p via viral transduction in VCaP and C4-2B cells (Fig. 3b). The effect of miR-210-3p on EMT in PCa cells was investigated and the result showed that silencing miR-210-3p converted the stick-like or long spindle shaped mesenchymal phenotype to an evident short spindle-shaped or cobblestone-like epithelial profile in PC-3 cells (Fig. 2i). As the epithelial cell phenotypes were predominant in the VCaP and C4-2B cells, we first treated in VCaP and C4-2B cells with TGF-β, which converted the evident short spindle-shaped or cobblestone-like epithelial profile to the stick-like or long spindle shaped mesenchymal phenotype (Fig. 3c). We further knocked down miR-210-3p expression in the TGF-β-treated VCaP and C4-2B cells and found that silencing miR-210-3p reversed the cell phenotypes in VCaP and C4-2B cells (Fig. 3c). Western blot analysis revealed that upregulating miR-210-3p reduced the expression of epithelial marker E-cadherin and enhanced the expression of mesenchymal marker vimentin and fibronectin in VCaP and C4-2B cells (Fig. 3d); conversely, silencing yielded an opposite effect on these EMT markers (Fig. 3d and Fig. 2j). Furthermore, invasion and migration assays were performed and the result indicated that upregulating miR-210-3p increased, while silencing miR-210-3p decreased the invasion and migration ability of PCa cells (Fig. 3e and f and Fig. 2k). These results indicate that miR-210-3p promotes the EMT, invasion and migration in PCa cells in vitro.

### miR-210-3p activates NF-kB signaling pathway in PCa cells

**Fig. 4:**
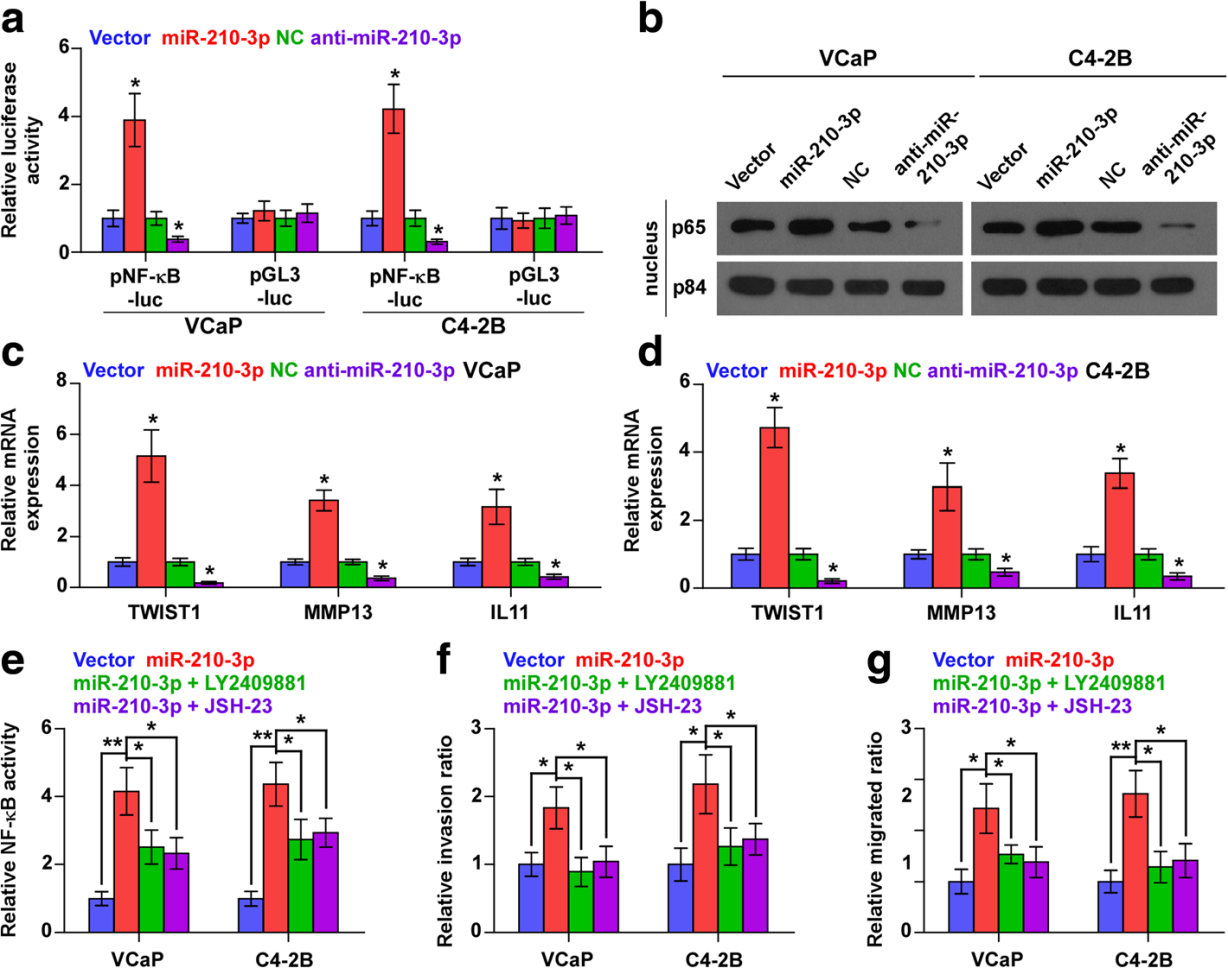
miR-210-3p activates NF-κB signaling pathway. **a** NF-κB transcriptional activity was assessed by luciferase reporter constructs in the indicated cells. Error bars represent the mean ± S.D. of three independent experiments. *P < 0.05. **b** Western blotting of nuclear NF-κB/p65 expression. The nuclear protein p84 was used as the nuclear protein marker. **c** and **d** Real-time PCR analysis of TWIST1, MMP13 and IL11 in the indicated cells. Transcript levels were normalized to *U6* expression. Error bars represent the mean ± S.D. of three independent experiments. *P < 0.05. **e** NF-κB signaling inhibitors LY2409881 (10 μM) and JSH-23 (10 μM) attenuated the stimulatory effect of miR-210-3p on NF-κB transcriptional activity in the indicated cells respectively. Error bars represent the mean ± s.d. of three independent experiments. *P < 0.05 and **P < 0.01. **f** NF-κB signaling inhibitors LY2409881 (10 μM) and JSH-23 (10 μM) attenuated the stimulatory effect of miR-210-3p on invasion ability in the indicated cells respectively. Error bars represent the mean ± s.d. of three independent experiments. *P < 0.05. **g** NF-κB signaling inhibitors LY2409881 (10 μM) and JSH-23 (10 μM) attenuated the stimulatory effect of miR-210-3p on migration ability in the indicated cells respectively. Error bars represent the mean ± s.d. of three independent experiments. *P < 0.05 and **P < 0.01

To investigate the underlying mechanism of the pro-bone metastasis role of miR-210-3p in PCa, a gene set enrichment analysis of miR-210-3p expression against the oncogenic signatures collection of the MSigDB was performed and the result showed that miR-210-3p overexpression significantly and positively correlated with NF-κB signaling (“JAIN_NF-κB_SIGNALING”) (Additional file 7: Figure S3a). These results suggest that miR-210-3p may regulate the NF-κB signaling pathways, which have been reported to promote bone metastasis in various types of cancers [18, 19]. As shown in Fig. 4a and Additional file 7: Figure S3b, we found that miR-210-3p overexpression significantly enhanced, while silencing miR-210-3p reduced NF-κB-dependent luciferase activity in PCa cells. Moreover, cellular fractionation and western blotting analysis revealed that overexpression of miR-210-3p enhanced, while silencing of miR-210-3p reduced nuclear accumulation of NF-κB/p65 (Fig. 4b and Additional file 7: Figure S3c). Real-time PCR analysis showed that upregulating miR-210-3p increased the expression levels of multiple NF-κB signaling downstream metastasis-related target genes including TWIST1, MMP13 and IL11 in PCa cells. By contrast, silencing miR-210-3p repressed these downstream genes in PCa cells (Fig. 4c and d and Additional file 7: Figure S3d). Thus, these results demonstrate that miR-210-3p activates NF-κB signaling pathway in PCa cells.

#### NF-κB activation mediates the pro-metastasis role of miR-210-3p in PCa cells

We further explored the functional significance of NF-κB signaling in the pro-metastasis role of miR-210-3p in PCa cells using NF-κB signaling inhibitors LY2409881 and JSH-23. As shown in Additional file 7: Figure S3e and f, LY2409881 and JSH-23 showed gradient inhibition of the NF-κB reporter activity in a dose-dependent manner in PCa cells. Notably, the stimulatory effect of miR-210-3p on NF-κB activity was attenuated by LY2409881 and JSH-23 (Fig. 4e). Moreover, inhibition of NF-κB signaling by LY2409881 and JSH-23 impaired the stimulatory effect of miR-210-3p overexpression on migration and invasion in PCa cells (Fig. 4f and g). These results suggest that NF-κB signaling activation is essential for the pro-metastasis role of miR-210-3p in PCa cells.

### miR-210-3p targets multiple negative regulators of NF-κB signaling

**Fig. 5:**
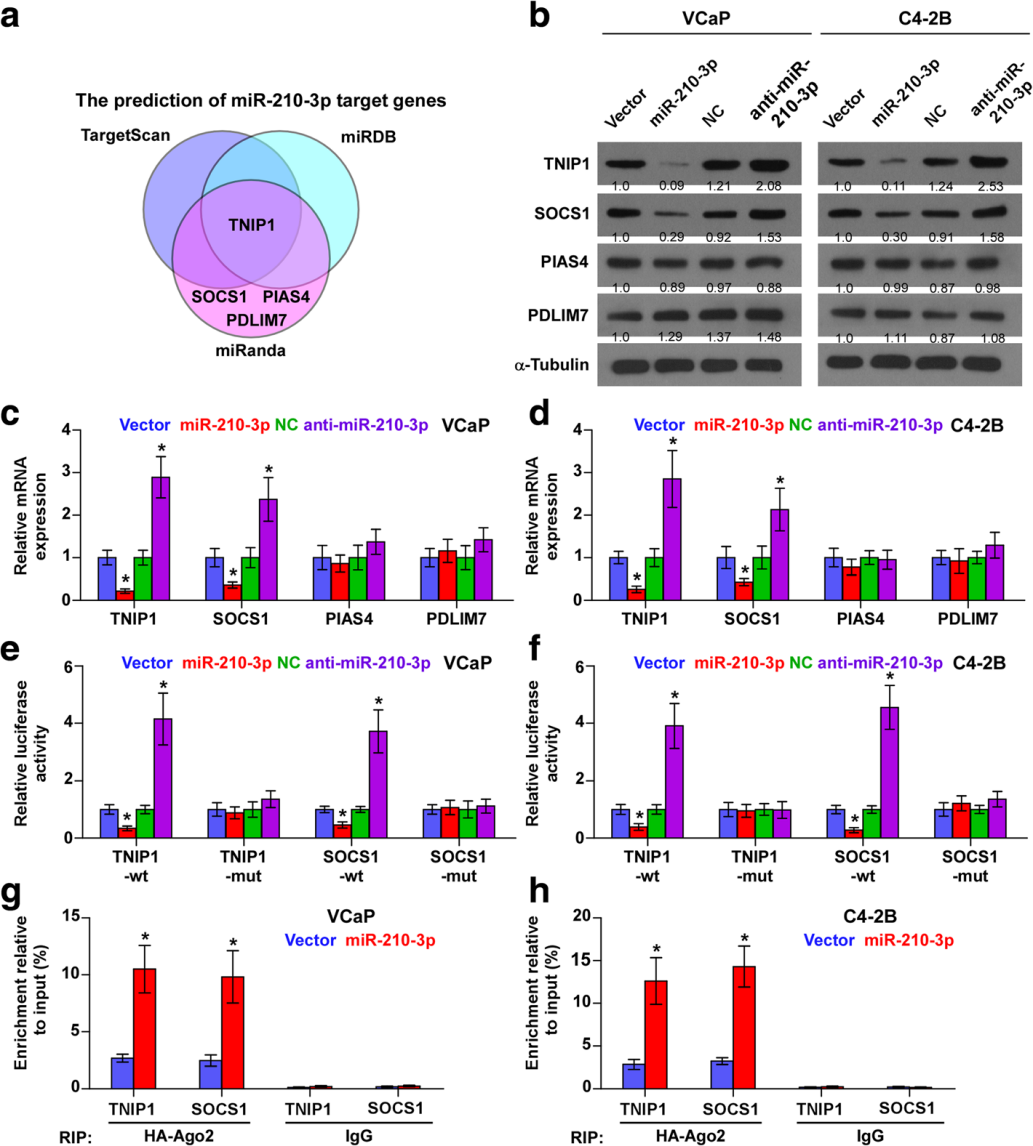
miR-210-3p targets multiple negative regulators of NF-κB signaling. **a** Predictive target genes of miR-210-3p from TargetScan, miRanda and miRDB. **b** Western blotting of TNIP1, SOCS1, PIAS4 and PDLIM7 expression in the indicated cells. α-Tubulin served as the loading control. Protein expression levels of TNIP1, SOCS1, PIAS4 and PDLIM7 were quantified by densitometry using Quantity One Software, and normalized to the corresponding expression levels of α-tubulin respectively. The sample 1 was used as a standard. **c** and **d** Real-time PCR analysis of TNIP1, SOCS1, PIAS4 and PDLIM7 expression in the indicated cells. Error bars represent the mean ± S.D. of three independent experiments. *P < 0.05. **e** and **f** Luciferase assay of cells transfected with pmirGLO-3’UTR reporter of TNIP1 and SOCS1 in the miR-210-3p overexpressing and silencing VCaP and C4-2B cells. ​*P < 0.05. **g** and **h** MiRNP IP assay showing the association between miR-210-3p and TNIP1 and SOCS1transcripts in PCa cells cells. Pulldown of IgG antibody served as the negative control​ *P < 0.05.

Using the publicly available algorithms TargetScan, miRanda and miRDB, we found that multiple negative regulators of NF-κB signaling, including TNIP1, SOCS1, PIAS4 and PDLIM7, may be potential targets of miR-210-3p (Fig. 5a and Additional file 8: Figure S4a). RT-PCR and western blotting analysis revealed that miR-210-3p overexpression reduced the expression levels of SOCS1 and TNIP1, but not of PIAS4 and PDLIM7 in PCa cells. In contrast, silencing miR-210-3p increased the expression levels of SOCS1 and TNIP1 (Fig. 5b-d and Additional file 8: Fig. S4b and c), indicating that miR-210-3p negatively regulated SOCS1 and TNIP1 in PCa cells. Moreover, luciferase assay revealed that upregulating miR-210-3p repressed, while silencing miR-210-3p elevated the reporter activity driven by the 3’UTRs of SOCS1 and TNIP1, but not by the mutant 3’UTR of SOCS1 and TNIP1 within the miR-210-3p–binding seed regions in PCa cells (Fig. 5e and f and Additional file 8: Fig. S4d). Moreover, microribonucleoprotein (miRNP) immunoprecipitation (IP) assay showed a direct association of miR-210-3p with SOCS1 and TNIP1 transcripts (Fig. 5g and h), which further demonstrated the direct repressive effects of miR-210-3p on SOCS1 and TNIP1. Furthermore, individual silencing of SOCS1 and TNIP1 reversed the repression of NF-κB activity by miR-210-3p silencing in PCa cells (Additional file 8: Figure S4e). Individual silencing of SOCS1 and TNIP1 rescued the repression of the invasive ability in miR-210-3p- silenced PCa cells (Additional file 8: Figure S4f). Taken together, our results suggest that miR-210-3p directly targets SOCS1 and TNIP1, resulting in constitutive activation of NF-κB signaling in PCa cells.

### Recurrent gains are the underlying mechanism responsible for miR-210-3p overexpression in a small portion of PCa patients

**Fig. 6:**
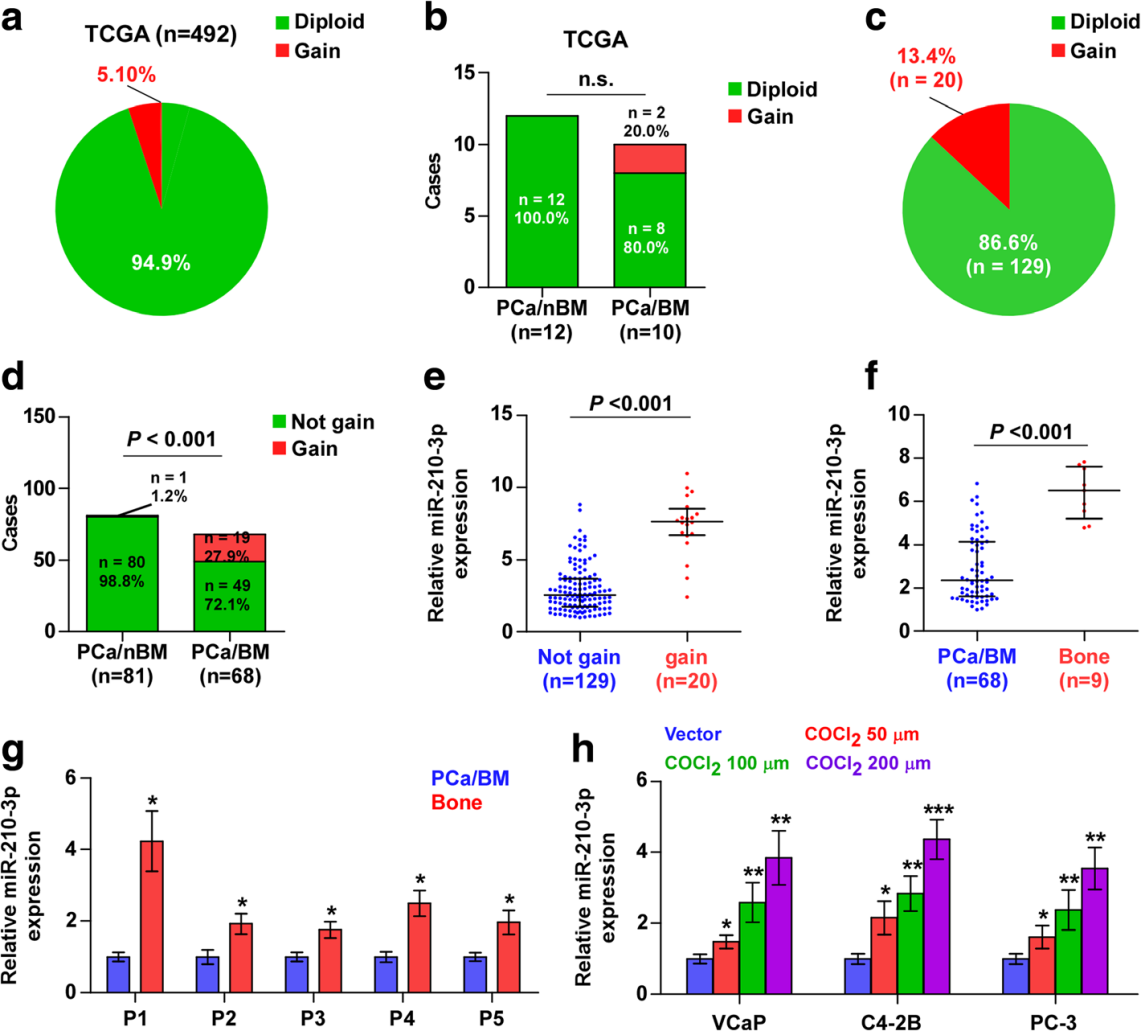
Recurrent gains are involved in miR-210-3p overexpression in PCa. **a** The percentage of miR-210-3p with gains in the PCa samples from TCGA. **b** Percentages and number of miR-210-3p samples with gains in PCa patients with different bone metastasis statues in TCGA dataset (n.s. = no significance). **c** The percentage of miR-210-3p with gains in our PCa samples. **d** Percentages and number of miR-210-3p samples with gains in PCa patients with different bone metastasis statues in our PCa samples. *P* < 0.001. **e** The average expression level of miR-210-3p in PCa tissues with gains was higher than those without gains in our PCa samples. Each bar represents the median values ± quartile values. *P* < 0.001. **f** miR-210-3p expression levels was markedly elevated in metastatic bone tissues compared with that in primary PCa tissues with bone metastasis (PCa/BM, *n* = 68; Bone, *n* = 9). *P* < 0.001. **g** miR-210-3p expression levels was differentially increased in metastatic bone tissues compared with that in corresponding paired priamy PCa tissues (*n* = 5). *P < 0.05. **h** The expression levels of miR-210-3p in the PCa cells under treatment of different concentration of COCl_2_. *P < 0.05, **P < 0.01 and ***P < 0.001

To further explore the underpinning mechanism of miR-210-3p overexpression in PCa tissues, we analyzed the PCa dataset from TCGA and found that recurrent gains (amplification) appeared in 5.1% of PCa tissues (Fig. 6a). Importantly, gains were observed in 2/10 bone metastatic PCa tissues, but were not observed in non-bone metastatic PCa tissues (Fig. 6b), indicating that miR-210-3p overexpression caused by gains may be implicated in the bone metastasis of PCa. We further measured the gain levels in our own PCa tissues and found that gains were found in 20/149 PCa tissues (approximately 13.4%) (Fig. 6c). Importantly, gains appeared in 19/68 bone metastatic PCa tissues (approximately 27.9%), but in 1 out of 81 non- bone metastatic PCa tissues (approximately 1.2%) (Fig. 6d). Furthermore, the expression level of miR-210-3p in PCa tissues with the gains was robustly higher than in those without gains (Fig. 6e). These results indicate that recurrent gains are implicated in miR-210-3p overexpression in a small portion of PCa patients.

### Hypoxic bone marrow microenvironment contributes to higher expression of miR-210-3p in metastatic bone tissues

Interestingly, a miRNA microarray from our previous study demonstrated that miR-210-3p was highly expressed in metastatic bone tissues than primary PCa tissues [39]. Real-time PCR analysis indicated that miR-210-3p expression in 9 individual metastatic bone tissues was significantly enhanced compared with that in 68 bone metastatic PCa tissues (Fig. 6f). Consistently, the analysis of the publicly available PCa datasets revealed that miR-210-3p expression in metastatic bone tissues was upregulated compared with that in primary PCa tissues (Additional file 9: Figure S5). Furthermore, the expression of miR-210-3p was measured in 5 paired PCa/bone tissues and we found that miR-210-3p expression was elevated in metastatic bone tissues compared with the matched primary PCa tissues (Fig. 6g).Taken together, these finding indicate that high expression of miR-210-3p may be involved in the whole process of bone metastasis in PCa, from escaping from primary PCa tissues to the development of secondary metastatic bone tumors.

To assess the mechanism underlying the higher expression of miR-210-3p in metastatic bone tissues compared with bone metastatic PCa tissues, numerous studies have reported that miR-210-3p is a direct target of hypoxia-inducible factor (HIF) [40, 41], and that the bone marrow microenvironment harbors extensive hypoxic regions characterized by abundant HIF [42–44]. Therefore, we further examined miR-210-3p expression in PCa cells under hypoxic conditions and found that miR-210-3p expression steadily increased with a gradient increase of the COCl2 concentration in PCa cells (Fig. 6h). Therefore, these findings indicate that hypoxic bone marrow microenvironment contributes to higher expression of miR-210-3p in metastatic bone tissues.

### Clinical correlation of miR-210-3p with TNIP1, SOCS1 and NF-κB activation in human PCa tissues

**Fig. 7:**
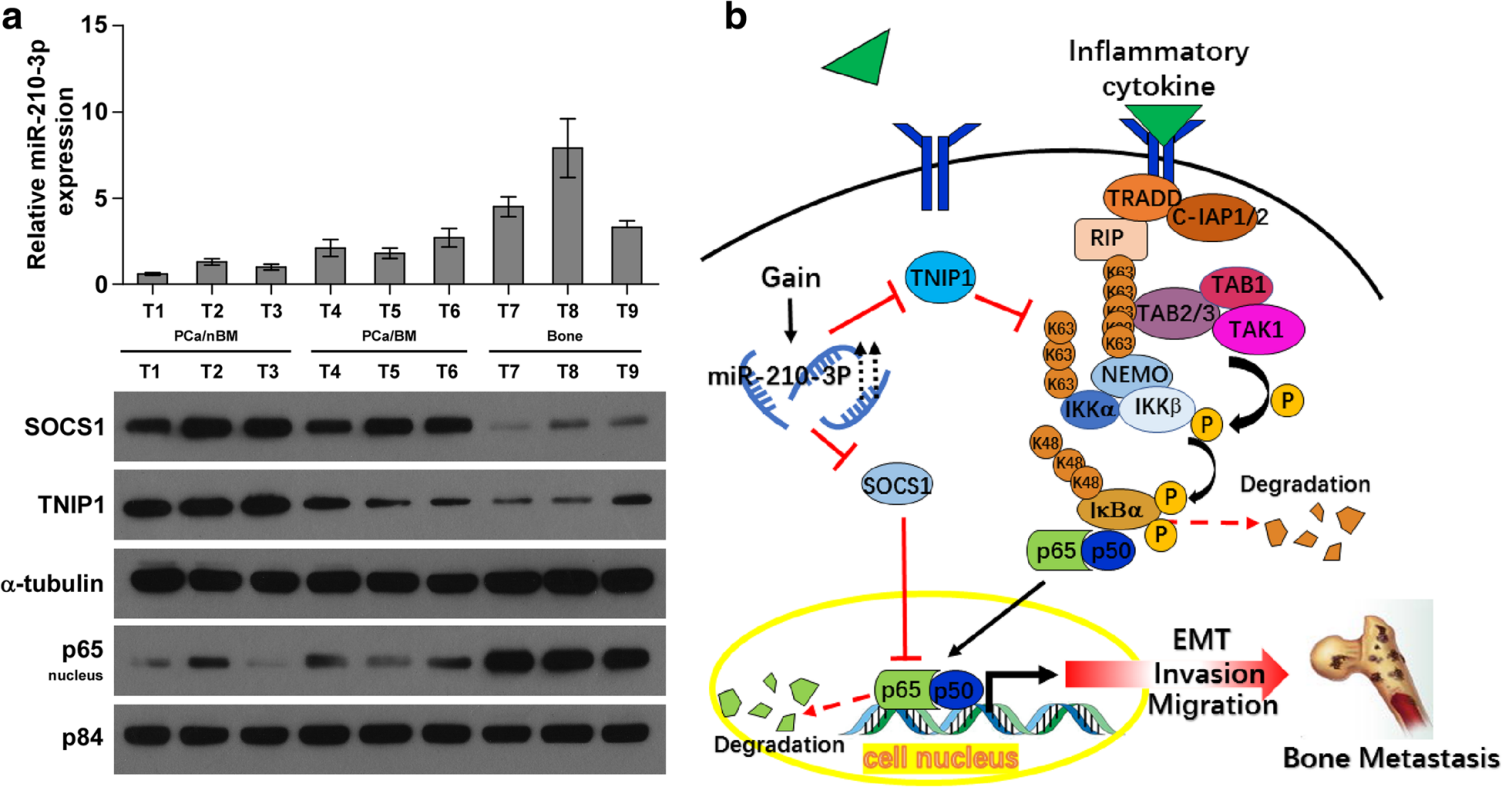
Clinical relevance of miR-210-3p with SOCS1, TNIP1 and NF-kB signaling activity in human PCa and bone tissues. **a** Analysis of miR-210-3p expression with SOCS1, TNIP1 and nuclear p65 in 3 bone metastatic PCa tissues, 3 non-bone metastatic PCa tissues and 3 metastatic bone tissues. U6 was used as the control for RNA loading. miR-210-3p expression levels were normalized to that miR-210-3p expression of sample one. Each bar represents the mean ± SD of three independent experiments. Loading controls were α-tubulin and p84 for the cytoplasmic and nuclear fractions. **b** Hypothetical model illustrating that constitutive activation of the NF-κB pathway by miR-210-3p epigenetic disruption of multiple negative feedback loops leads to bone metastasis of PCa

To further investigate the clinical significance of miR-210-3p-induced TNIP1 and SOCS1 downregulation and the subsequent activation of NF-κB signaling in PCa tissues, miR-210-3p expression and the protein expression levels of TNIP1, SOCS1 and nuclear p65 were examined. As shown in Fig. 7a, miR-210-3p and nuclear p65 expression in bone metastatic PCa tissues (T4–6) was elevated compared with that in non-bone metastatic PCa tissues (T1–3)and further increased in metastatic bone tissues (T7–9). Conversely, protein expression of SOCS1 and TNIP1 exhibited an opposite pattern (Fig. 7a). Pearson analysis revealed that miR-210-3p expression inversely correlated with SOCS1 (Additional file 10: Figure S6a. *r* = −0.682, *P* < 0.05) and TNIP1 (Additional file 10: Figure S6b. *r* = −0.798, *P* < 0.05), but strongly correlated with nuclear p65 expression (Additional file 10: Figure S6c. *r* = 0.769, *P* < 0.05). Taken together, our results indicate that overexpression of miR-210-3p activates NF-κB signaling by inhibiting TNIP1 and SOCS1, resulting in the bone metastasis of PCa (Fig. 7b).

## Discussion

The key findings of the current study present novel insights into the critical role of miR-210-3p in the sustained activation of NF-κB signaling, which further promotes bone metastasis of PCa. Here, we reported that miR-210-3p expression was elevated in bone metastatic PCa tissues, which was caused by recurrent gains, and high expression of miR-210-3p correlated with PSA levels, Gleason grade and bone metastasis status in PCa patients. Our results further indicate that miR-210-3p activates NF-κB signaling in PCa cells via directly targeting SOCS1 and TNIP1, resulting in the development of PCa bone metastasis. Therefore, our results uncover a novel mechanism by which miR-210-3p sustains constitutive activation of NF-κB signaling, elucidating the oncogenic function of miR-210-3p in bone metastasis of PCa.

Extensive research efforts have shown that NF-κB signaling was constitutively activated in several types of human cancer, which was significantly associated with the tumor progression and metastasis [15, 17]. For example, in glioma, activation of NF-κB signaling was crucial for the promotion of glioma cell invasion and migration [45, 46]; in addition, a study by Helbig and colleagues has noted that expression of chemokine receptor CXCR4 was induced by activation of NF-κB signaling, which promoted the migration and metastasis of breast cancer cells [47]. Emerging literatures have shown that NF-κB signaling plays an important role in the bone metastasis of cancers [18, 19]. Park and colleagues reported that constitutive NF-κB activity in breast cancer cells was crucial for the bone resorption characteristic of osteolytic bone metastasis. The identified gene mediated osteolytic bone metastasis of breast cancer was a key target of NF-κB signaling: granulocyte macrophage-colony stimulating factor (GM-CSF) promoted osteolytic bone metastasis of breast cancer cells by stimulating osteoclast development [20]. Importantly, Chen et al. reported that NF-κB activation also played a pivotal role in the development of PCa bone metastasis [19]. However, the underlying mechanism responsible for constitutive activation of NF-κB signaling in the bone metastasis of PCa remains largely unknown. Here, we report that miR-210-3p activated NF-κB signaling through directly targeting SOCS1 and TNIP1 in PCa cells, which promoted the development of bone metastasis of PCa. Furthermore, NF-κB signaling activity repressed by the specific inhibitors attenuated the stimulatory role of upregulating miR-210-3p on invasion and migration of PCa cells. Taken together, our results indicate that high expression of miR-210-3p constitutively activates NF-κB signaling, which is essential for bone metastasis of PCa.

Numerous lines of evidence have indicated that deficiencies or downregulation of negative regulators of the NF-κB signaling pathway could lead to constitutive activation of NF-κB signaling, which further promoted tumor progression and metastasis [48–50]. Multiple well-known negative regulators of NF-κB signaling, such as CYLD, TNIPs and A20, have been reported to restrict the activity of NF-κB signaling via different negative feedback mechanisms. TNIPs, which were found to exert functions by linking A20 to NEMO and accelerate A20-mediated NF-κB signaling activity inhibition through deubiquitination of NEMO, have been reported to participate in the inhibition of NF-κB signaling activity [51]. On the other hand, extensive crosstalk between inhibitors or negative regulators of other signaling pathways, such as JAK/STAT signaling, and NF-κB signaling activity were broadly reported. For example, PIAS4, a member of the PIAS (protein inhibitor of activated STAT) protein family, which was originally identified as inhibitors of the STAT proteins, has been reported to be an important repressor of NF-κB signaling activation via regulating TRIF-induced NF-κB signaling activation [52, 53]. Moreover, STAT3 signaling inhibitor suppressor of cytokine signaling (SOCS1) has been reported to promote the degradation of the DNA-bound p65 protein, leading to the suppression of NF-κB activity [54–57]. However, how cancer cells simultaneously take priority over these feedback loops in PCa remains obscure. In this study, our results demonstrated that high expression of miR-210-3p constitutively activated NF-κB signaling via simultaneously suppressing negative regulators of NF-κB signaling TNIP1 and SOCS1, resulting in the bone metastasis of PCa. Therefore, our finding uncover a novel mechanism by which miR-210-3p disrupts the negative feedback loops of NF-κB signaling in PCa cells, which results in constitutive activation of NF-κB signaling, supporting the notion that NF-κB signaling contributes to the bone metastasis of PCa.

Hypoxia has been identified as a critical contributor to the tumor development, progression and metastasis, where the hypoxic environment exerts its functions via inducing the production of hypoxia inducible factor (HIF), which then transcriptionally activates a wide array of downstream molecules for adaptation to the hypoxic condition [58, 59]. The bone marrow microenvironment possesses extensive hypoxic regions [42, 43] that are characterized by abundant HIF-1α staining and HIF target proteins including MCT4 and Glut1 [44]. It’s notable that the hypoxic microenvironment of the bone marrow is conductive to subsequent bone colonization of cancer cells, and therapies targeting HIF/HIF targets has potential value in the prevention of bone colonization [60–62]. Furthermore, accumulating studies revealed that miRNAs are emerging as a novel class targets of hypoxia-responsive molecules [63, 64]. It’s worth noting that miR-210-3p has been broadly demonstrated to be a direct target of HIF-1α in a variety of tumor cells [40, 41]. Therefore, it’s conceivable that miR-210-3p expression in bone tissues will be elevated compared with primary PCa tissues due to the inducible effects of abundant HIF within the hypoxic bone marrow microenvironment. Indeed, our results revealed that miR-210-3p expression in metastatic bone tissues was upregulated compared with primary PCa tissues. Furthermore, several lines of evidence reported that activation of NF-κB signaling promoted the attachment and growth of cancer cells in bone via upregulating multiple osteoclastogenesis-associated genes, including RANKL, PTHrP and GM-CSF, resulting in osteolytic bone metastasis of cancer [20, 65]. In this study, our results demonstrated that overexpression of miR-210-3p augmented the NF-κB signaling activity via targeting TNIP1 and SOCS1 in PCa cells. Therefore, these findings suggest that a hypoxic bone microenvironment promotes bone colonization of cancer cells to bone via activation of miR-210-3p/ NF-κB signaling axis, which contributes to the development of bone metastatic disease in PCa.

Several studies have indicated that miR-210-3p was upregulated in multiple human cancers and that high expression of miR-210-3p promoted cancer cell invasion and metastasis via different mechanisms and predicted poor survival [40, 41, 66–68]. Furthermore, recent literatures have identified miR-210-3p as a serum marker in many types of cancer, which will facilitate the early detection of metastatic tumors [68, 69]. Notably, Tewari and the colleagues reported that miR-210-3p was dramatically elevated in the serum of metastatic castration resistant prostate cancer patients compared with healthy controls, indicating that miR-210-3p was involved in the metastasis of PCa [41]. Moreover, a study by Taddei showed that hypoxia-induced miR-210 in fibroblasts enhanced the senescence-associated features, which promoted PCa aggressiveness by inducing EMT and by secreting energy-rich compounds to support PCa cell growth [70]. However, the biological roles and clinical significance of miR-210-3p in bone metastasis of PCa remains largely unknown. In this study, our results revealed that miR-210-3p was elevated in human bone metastatic PCa tissues and cells. High expression of miR-210-3p correlated with serum PSA level, Gleason grade and distant bone metastasis status in PCa patients. Moreover, our results revealed that miR-210-3p activated NF-κB signaling via targeting TNIP1 and SOCS1, which further promoted the EMT, invasion, migration and bone metastasis of PCa cells in vitro and in vivo. Furthermore, our finding demonstrated that recurrent gains are the underlying mechanism contributing to miR-210-3p overexpression in bone metastatic PCa tissues. Collectively, our findings indicate that miR-210-3p plays an important role in the bone metastasis of PCa.

## Conclusions

In summary, our results demonstrate that upregulation of miR-210-3p caused by recurrent gains activates NF-κB signaling pathway, which further promotes bone metastasis in PCa. Thus, the findings of this current study improve our understanding of the molecular mechanisms underlying constitutive activation of NF-κB in bone metastasis of PCa, and provide novel insights into the development of anti-bone metastasis therapeutic strategies for PCa via silencing miR-210-3p.

## Additional files

Additional file 1: Table S1. A list of primers used in the reactions for clone PCR. (PDF 6 kb)

Additional file 2: Table S2. A list of primers used in the reactions for real-time RT-PCR. (PDF 9 kb)

Additional file 3: Table S3. The clinicopathological characteristics in 149 patients with prostate cancer. (PDF 54 kb)

Additional file 4: Figure S1. miR-210-3p expression is upregulated in bone metastatic PCa tissues and cells. **(a)** Percentages and number of samples showed high or low miR-210-3p expression in our PCa patients with different bone metastasis. *P* < 0.001. **(b)** miR-210-3p expression was elevated in PCa cells compared with that in stromal cells in GSE17321 dataset. *P < 0.05. (PDF 62 kb)

Additional file 5: Table S4. The relationship between miR-210-3p and clinicopathological characteristics in 149 patients with prostate cancer. (PDF 58 kb)

Additional file 6: Figure S2. Silencing miR-210-3p repressed EMT, invasion and migration in PC-3 cells in vitro. Real-time PCR analysis of miR-210-3p expression in PC-3 cells transduced with antagomiR-210-3p compared to controls. Transcript levels were normalized by *U6* expression. Error bars represent the mean ± s.d. of three independent experiments. *P < 0.05.

Additional file 7: Figure S3. Silencing miR-210-3p inhibits NF-κB signaling activity in PC-3 cells. **(a)** Gene set enrichment analysis (GSEA) revealed that miR-210-3p expression significantly and positively correlated with the NF-κB signaling. **(b)** NF-κB transcriptional activity was repressed by silencing miR-210-3p in the indicated PC-3 cells. Error bars represent the mean ± S.D. of three independent experiments. *P < 0.05. **(c)** Western blotting of nuclear NF-κB/p65 expression. The nuclear protein p84 was used as the nuclear protein marker. **(d)** Real-time PCR analysis of TWIST1, MMP13 and IL11 in the indicated cells. Error bars represent the mean ± S.D. of three independent experiments. *P < 0.05. **(e** and **f)** NF-κB signaling inhibitors LY2409881 and JSH-23 inhibited the NF-κB transcriptional activity in a dose-dependent manner in the indicated cells. Error bars represent the mean ± S.D. of three independent experiments. *P < 0.05, **P < 0.01 and ***P < 0.001. (PDF 128 kb)

Additional file 8: Figure S4. miR-210-3p targets multiple negative regulators of NF-κB signaling. **(a)** Predicted miR-210-3p targeting sequence and mutant sequences in 3’UTR s of SOCS1 and TNIP1. **(b)** Real-time PCR analysis of TNIP1, SOCS1, PIAS4 and PDLIM7 expression in the indicated cells. Error bars represent the mean ± S.D. of three independent experiments. *P < 0.05. **(c)** Western blotting of TNIP1, SOCS1, PIAS4 and PDLIM7 expression in the indicated cells. α-Tubulin served as the loading control. **(d)** Luciferase assay of cells transfected with pmirGLO-3’UTR reporter of TNIP1 and SOCS1 in the miR-210-3p silencing PC-3 cells. *P < 0.05. **(e** and **f)** Individual silencing of TNIP1 and SOCS1 rescued the NF-κB activity **(e)** and invasion **(f)** abilities repressed by miR-210-3p silencing in PCa cells. *P < 0.05 and **P < 0.01. (PDF 185 kb)

Additional file 9: Figure S5. miR-210-3p expression levels was markedly elevated in metastatic bone tissues compared with that in primary PCa tissues with bone metastasis (BM, *n* = 6; Bone, *n* = 7). *P < 0.05. (PDF 28 kb)

Additional file 10: Figure S6. Clinical correaltion of miR-210-3p with SOCS1, TNIP1 and nuclear p65 in human PCa and bone tissues. (**a-c**) Correlation between miR-210-3p levels and SOCS1, TNIP1 and nuclear p65 expression in PCa and bone tissues.The expression levels of SOCS1, TNIP1 and nuclear p65 were quantified by densitometry using Quantity One Software, and normalized to the levels of α-tubulin and p84, respectively. The sample 1 was used as a standard. The relative expressions of miR-210-3p and these proteins were used to perform the correlation analysis. (PDF 88 kb)

## Abbreviations

EMT: Epithelial-mesenchymal transition
ERBB2: Erb-B2 Receptor Tyrosine Kinase 2
H&E: Hematoxylin and Eosin Stain
IL11: Interleukin 11
miR-210-3p: miRNA-210-3p
MMP13: Matrix metallopeptidase 13
NF-κB: Nuclear factor kappa-light-chain-enhancer of activated B cells
PCR: Polymerase Chain Reaction
PDLIM7: PDZ and LIM domain 7
PIAS4: Protein inhibitor of activated STAT 4
SNAIL1: Snail family transcriptional repressor 1
SOCS1: suppressor of cytokine signaling 1
TCGA: The Cancer Genome Atlas
TNIP1: TNFAIP3 interacting protein 1
TWIST1: Twist family bHLH transcription factor 1
ZEB1/2: Zinc finger E-box binding homeobox 1/2

## References

[1] Nelson WG, De Marzo AM, Isaacs WB (2003). Prostate cancer. N Engl J Med.

[2] Carlin BI, Andriole GL (2000). The natural history, skeletal complications, and management of bone metastases in patients with prostate carcinoma. Cancer.

[3] Bubendorf L, Schopfer A, Wagner U, Sauter G, Moch H, Willi N, Gasser TC, Mihatsch MJ (2000). Metastatic patterns of prostate cancer: an autopsy study of 1,589 patients. Hum Pathol.

[4] Thiery JP, Acloque H, Huang RY, Nieto MA (2009). Epithelial-mesenchymal transitions in development and disease. Cell.

[5] Kalluri R, Weinberg RA (2009). The basics of epithelial-mesenchymal transition. J Clin Invest.

[6] Miettinen PJ, Ebner R, Lopez AR, Derynck R (1994). TGF-beta induced transdifferentiation of mammary epithelial cells to mesenchymal cells: involvement of type I receptors. J Cell Biol.

[7] Wu Y, Deng J, Rychahou PG, Qiu S, Evers BM, Zhou BP (2009). Stabilization of snail by NF-kappaB is required for inflammation-induced cell migration and invasion. Cancer Cell.

[8] Wang M, Ren D, Guo W, Huang S, Wang Z, Li Q, Du H, Song L, Peng X (2016). N-cadherin promotes epithelial-mesenchymal transition and cancer stem cell-like traits via ErbB signaling in prostate cancer cells. Int J Oncol.

[9] Masszi A, Fan L, Rosivall L, McCulloch CA, Rotstein OD, Mucsi I, Kapus A (2004). Integrity of cell-cell contacts is a critical regulator of TGF-beta 1-induced epithelial-to-myofibroblast transition: role for beta-catenin. Am J Pathol.

[10] Tan EJ, Thuault S, Caja L, Carletti T, Heldin CH, Moustakas A (2012). Regulation of transcription factor twist expression by the DNA architectural protein high mobility group A2 during epithelial-to-mesenchymal transition. J Biol Chem.

[11] Thuault S, Tan EJ, Peinado H, Cano A, Heldin CH, Moustakas A (2008). HMGA2 and Smads co-regulate SNAIL1 expression during induction of epithelial-to-mesenchymal transition. J Biol Chem.

[12] Huber MA, Azoitei N, Baumann B, Grunert S, Sommer A, Pehamberger H, Kraut N, Beug H, Wirth T (2004). NF-kappaB is essential for epithelial-mesenchymal transition and metastasis in a model of breast cancer progression. J Clin Invest.

[13] Song R, Song H, Liang Y, Yin D, Zhang H, Zheng T, Wang J, Lu Z, Song X, Pei T (2014). Reciprocal activation between ATPase inhibitory factor 1 and NF-kappaB drives hepatocellular carcinoma angiogenesis and metastasis. Hepatology.

[14] Sen R, Baltimore D (1986). Multiple nuclear factors interact with the immunoglobulin enhancer sequences. Cell.

[15] Karin M, Greten FR (2005). NF-kappaB: linking inflammation and immunity to cancer development and progression. Nat Rev Immunol.

[16] Hayden MS, Ghosh S (2008). Shared principles in NF-kappaB signaling. Cell.

[17] Hoesel B, Schmid JA (2013). The complexity of NF-kappaB signaling in inflammation and cancer. Mol Cancer.

[18] Burnett RM, Craven KE, Krishnamurthy P, Goswami CP, Badve S, Crooks P, Mathews WP, Bhat-Nakshatri P, Nakshatri H (2015). Organ-specific adaptive signaling pathway activation in metastatic breast cancer cells. Oncotarget.

[19] Chen PC, Cheng HC, Tang CH (2013). CCN3 promotes prostate cancer bone metastasis by modulating the tumor-bone microenvironment through RANKL-dependent pathway. Carcinogenesis.

[20] Park BK, Zhang H, Zeng Q, Dai J, Keller ET, Giordano T, Gu K, Shah V, Pei L, Zarbo RJ (2007). NF-kappaB in breast cancer cells promotes osteolytic bone metastasis by inducing osteoclastogenesis via GM-CSF. Nat Med.

[21] Nguyen DP, Li J, Yadav SS, Tewari AK (2014). Recent insights into NF-kappaB signalling pathways and the link between inflammation and prostate cancer. BJU Int.

[22] Bartel DP (2009). MicroRNAs: target recognition and regulatory functions. Cell.

[23] Velu VK, Ramesh R, Srinivasan AR (2012). Circulating MicroRNAs as biomarkers in health and disease. J Clin Diagn Res.

[24] Khew-Goodall Y, Goodall GJ (2010). Myc-modulated miR-9 makes more metastases. Nat Cell Biol.

[25] Ma L, Young J, Prabhala H, Pan E, Mestdagh P, Muth D, Teruya-Feldstein J, Reinhardt F, Onder TT (2010). Valastyan S, et al: miR-9, a MYC/MYCN-activated microRNA, regulates E-cadherin and cancer metastasis. Nat Cell Biol.

[26] Colden M, Dar AA, Saini S, Dahiya PV, Shahryari V, Yamamura S, Tanaka Y, Stein G, Dahiya R, Majid S (2017). MicroRNA-466 inhibits tumor growth and bone metastasis in prostate cancer by direct regulation of osteogenic transcription factor RUNX2. Cell Death Dis.

[27] Siu MK, Tsai YC, Chang YS, Yin JJ, Suau F, Chen WY, Liu YN (2015). Transforming growth factor-beta promotes prostate bone metastasis through induction of microRNA-96 and activation of the mTOR pathway. Oncogene.

[28] Ren D, Wang M, Guo W, Huang S, Wang Z, Zhao X, Du H, Song L, Peng X (2014). Double-negative feedback loop between ZEB2 and miR-145 regulates epithelial-mesenchymal transition and stem cell properties in prostate cancer cells. Cell Tissue Res.

[29] Ren D, Wang M, Guo W, Zhao X, Tu X, Huang S, Zou X, Peng X (2013). Wild-type p53 suppresses the epithelial-mesenchymal transition and stemness in PC-3 prostate cancer cells by modulating miR145. Int J Oncol.

[30] Guo W, Ren D, Chen X, Tu X, Huang S, Wang M, Song L, Zou X, Peng X (2013). HEF1 promotes epithelial mesenchymal transition and bone invasion in prostate cancer under the regulation of microRNA-145. J Cell Biochem.

[31] Wu TT, Sikes RA, Cui Q, Thalmann GN, Kao C, Murphy CF, Yang H, Zhau HE, Balian G, Chung LW (1998). Establishing human prostate cancer cell xenografts in bone: induction of osteoblastic reaction by prostate-specific antigen-producing tumors in athymic and SCID/bg mice using LNCaP and lineage-derived metastatic sublines. Int J Cancer.

[32] Cui XY, Skretting G, Jing Y, Sun H, Sandset PM, Sun L (2013). Hypoxia influences stem cell-like properties in multidrug resistant K562 leukemic cells. Blood Cells Mol Dis.

[33] Piret JP, Mottet D, Raes M, Michiels C (2002). CoCl2, a chemical inducer of hypoxia-inducible factor-1, and hypoxia reduce apoptotic cell death in hepatoma cell line HepG2. Ann N Y Acad Sci.

[34] Mermel CH, Schumacher SE, Hill B, Meyerson ML, Beroukhim R, Getz G (2011). GISTIC2.0 facilitates sensitive and confident localization of the targets of focal somatic copy-number alteration in human cancers. Genome Biol.

[35] Zhang X, Ren D, Guo L, Wang L, Wu S, Lin C, Ye L, Zhu J, Li J, Song L (2017). Thymosin beta 10 is a key regulator of tumorigenesis and metastasis and a novel serum marker in breast cancer. Breast Cancer Res.

[36] Li X, Liu F, Lin B, Luo H, Liu M, Wu J, Li C, Li R, Zhang X, Zhou K, Ren D: miR150 inhibits proliferation and tumorigenicity via retarding G1/S phase transition in nasopharyngeal carcinoma. Int J Oncol 2017 doi: 10.3892/ijo.2017.3909. 10.3892/ijo.2017.3909 PMC5363880 28350089

[37] Wang M, Ren D, Guo W, Wang Z, Huang S, Du H, Song L, Peng X (2014). Loss of miR-100 enhances migration, invasion, epithelial-mesenchymal transition and stemness properties in prostate cancer cells through targeting Argonaute 2. Int J Oncol.

[38] Liu YN, Yin JJ, Abou-Kheir W, Hynes PG, Casey OM, Fang L, Yi M, Stephens RM, Seng V, Sheppard-Tillman H (2013). MiR-1 and miR-200 inhibit EMT via slug-dependent and tumorigenesis via slug-independent mechanisms. Oncogene.

[39] Peng X, Guo W, Liu T, Wang X, Tu X, Xiong D, Chen S, Lai Y, Du H, Chen G (2011). Identification of miRs-143 and -145 that is associated with bone metastasis of prostate cancer and involved in the regulation of EMT. PLoS One.

[40] Ding L, Zhao L, Chen W, Liu T, Li Z (2015). Li X: miR-210, a modulator of hypoxia-induced epithelial-mesenchymal transition in ovarian cancer cell. Int J Clin Exp Med.

[41] Cheng HH, Mitchell PS, Kroh EM, Dowell AE, Chery L, Siddiqui J, Nelson PS, Vessella RL, Knudsen BS, Chinnaiyan AM (2013). Circulating microRNA profiling identifies a subset of metastatic prostate cancer patients with evidence of cancer-associated hypoxia. PLoS One.

[42] Parmar K, Mauch P, Vergilio JA, Sackstein R, Down JD (2007). Distribution of hematopoietic stem cells in the bone marrow according to regional hypoxia. Proc Natl Acad Sci U S A.

[43] Rankin EB, Giaccia AJ, Schipani E (2011). A central role for hypoxic signaling in cartilage, bone, and hematopoiesis. Curr Osteoporos Rep.

[44] Kusumbe AP, Ramasamy SK, Adams RH (2014). Coupling of angiogenesis and osteogenesis by a specific vessel subtype in bone. Nature.

[45] Jiang L, Lin C, Song L, Wu J, Chen B, Ying Z, Fang L, Yan X, He M, Li J, Li M (2012). MicroRNA-30e* promotes human glioma cell invasiveness in an orthotopic xenotransplantation model by disrupting the NF-kappaB/IkappaBalpha negative feedback loop. J Clin Invest.

[46] Jiang L, Wu J, Yang Y, Liu L, Song L, Li J, Li M (2012). Bmi-1 promotes the aggressiveness of glioma via activating the NF-kappaB/MMP-9 signaling pathway. BMC Cancer.

[47] Helbig G, Christopherson KW, Bhat-Nakshatri P, Kumar S, Kishimoto H, Miller KD, Broxmeyer HE, Nakshatri H (2003). NF-kappaB promotes breast cancer cell migration and metastasis by inducing the expression of the chemokine receptor CXCR4. J Biol Chem.

[48] Sun SC (2010). CYLD: a tumor suppressor deubiquitinase regulating NF-kappaB activation and diverse biological processes. Cell Death Differ.

[49] Espinosa L, Cathelin S, D'Altri T, Trimarchi T, Statnikov A, Guiu J, Rodilla V, Ingles-Esteve J, Nomdedeu J, Bellosillo B (2010). The notch/Hes1 pathway sustains NF-kappaB activation through CYLD repression in T cell leukemia. Cancer Cell.

[50] Hellerbrand C, Bumes E, Bataille F, Dietmaier W, Massoumi R, Bosserhoff AK (2007). Reduced expression of CYLD in human colon and hepatocellular carcinomas. Carcinogenesis.

[51] Verstrepen L, Carpentier I, Verhelst K, Beyaert R (2009). ABINs: A20 binding inhibitors of NF-kappa B and apoptosis signaling. Biochem Pharmacol.

[52] Zhang J, Xu LG, Han KJ, Wei X, Shu HB (2004). PIASy represses TRIF-induced ISRE and NF-kappaB activation but not apoptosis. FEBS Lett.

[53] Tahk S, Liu B, Chernishof V, Wong KA, Wu H, Shuai K (2007). Control of specificity and magnitude of NF-kappa B and STAT1-mediated gene activation through PIASy and PIAS1 cooperation. Proc Natl Acad Sci U S A.

[54] Ben-Neriah Y (2003). Pinning NF-kappaB to the nucleus. Mol Cell.

[55] Park SH, Kim KE, Hwang HY, Kim TY (2003). Regulatory effect of SOCS on NF-kappaB activity in murine monocytes/macrophages. DNA Cell Biol.

[56] Gingras S, Parganas E, de Pauw A, Ihle JN, Murray PJ (2004). Re-examination of the role of suppressor of cytokine signaling 1 (SOCS1) in the regulation of toll-like receptor signaling. J Biol Chem.

[57] Baig MS, Zaichick SV, Mao M, de Abreu AL, Bakhshi FR, Hart PC, Saqib U, Deng J, Chatterjee S, Block ML (2015). NOS1-derived nitric oxide promotes NF-kappaB transcriptional activity through inhibition of suppressor of cytokine signaling-1. J Exp Med.

[58] Harris AL (2002). Hypoxia--a key regulatory factor in tumour growth. Nat Rev Cancer.

[59] Tsai YP, Wu KJ (2012). Hypoxia-regulated target genes implicated in tumor metastasis. J Biomed Sci.

[60] Zolnierek J, Nurzynski P, Langiewicz P, Oborska S, Wasko-Grabowska A, Kuszatal E, Obrocka B, Szczylik C (2010). Efficacy of targeted therapy in patients with renal cell carcinoma with pre-existing or new bone metastases. J Cancer Res Clin Oncol.

[61] Molina AM, Jia X, Feldman DR, Hsieh JJ, Ginsberg MS, Velasco S, Patil S, Motzer RJ (2013). Long-term response to sunitinib therapy for metastatic renal cell carcinoma. Clin Genitourin Cancer.

[62] Catena R, Luis-Ravelo D, Anton I, Zandueta C, Salazar-Colocho P, Larzabal L, Calvo A, Lecanda F (2011). PDGFR signaling blockade in marrow stroma impairs lung cancer bone metastasis. Cancer Res.

[63] Ivan M, Harris AL, Martelli F, Kulshreshtha R (2008). Hypoxia response and microRNAs: no longer two separate worlds. J Cell Mol Med.

[64] Greco S, Martelli F (2014). MicroRNAs in hypoxia response. Antioxid Redox Signal.

[65] Jin R, Sterling JA, Edwards JR, DeGraff DJ, Lee C, Park SI, Matusik RJ (2013). Activation of NF-kappa B signaling promotes growth of prostate cancer cells in bone. PLoS One.

[66] Kai AK, Chan LK, Lo RC, Lee JM, Wong CC, Wong JC, Ng IO (2016). Down-regulation of TIMP2 by HIF-1alpha/miR-210/HIF-3alpha regulatory feedback circuit enhances cancer metastasis in hepatocellular carcinoma. Hepatology.

[67] Madhavan D, Peng C, Wallwiener M, Zucknick M, Nees J, Schott S, Rudolph A, Riethdorf S, Trumpp A, Pantel K (2016). Circulating miRNAs with prognostic value in metastatic breast cancer and for early detection of metastasis. Carcinogenesis.

[68] Ono S, Oyama T, Lam S, Chong K, Foshag LJ, Hoon DS (2015). A direct plasma assay of circulating microRNA-210 of hypoxia can identify early systemic metastasis recurrence in melanoma patients. Oncotarget.

[69] Chen J, Wang W, Zhang Y, Chen Y, Hu T (2014). Predicting distant metastasis and chemoresistance using plasma miRNAs. Med Oncol.

[70] Taddei ML, Cavallini L, Comito G, Giannoni E, Folini M, Marini A, Gandellini P, Morandi A, Pintus G, Raspollini MR (2014). Senescent stroma promotes prostate cancer progression: the role of miR-210. Mol Oncol.

