# Supplementary material for: Oncogenic miR-210-3p promotes prostate cancer cell EMT and bone metastasis via NF-κB signaling pathway
Source: Mol Cancer. 2017 Jul 10;16:117. doi: 10.1186/s12943-017-0688-6 (PMC5504657; doi:10.1186/s12943-017-0688-6)
Supplement: Supplementary file 1 — A list of primers used in the reactions for clone PCR. (PDF 6 kb) [file 12943_2017_688_MOESM1_ESM.pdf]

Table S1. A list of primers used in the reactions for clone PCR.

**Used for subcloning and plasmid construction:**	
miR-210-3p-clone-F	GGGACCAGGTCATTTGCATACG
miR-210-3p-clone-R	CCCGAATGATTTCGCTTACCC
SOCS1-3UTR-149nt-clone-F	TCGAGCTGCTGGAGCACTAC
SOCS1-3UTR-261nt-clone-R	CAGGTCCTGGCTCCAGATAC
TNIP1-3UTR-167nt-clone-F	CTCAGCTGGATGAGAGGCTGAG
TNIP1-3UTR-774nt-clone-R	AGCCACCCTAATCTGGGCTTC
