# Supplementary material for: Oncogenic miR-210-3p promotes prostate cancer cell EMT and bone metastasis via NF-κB signaling pathway
Source: Mol Cancer. 2017 Jul 10;16:117. doi: 10.1186/s12943-017-0688-6 (PMC5504657; doi:10.1186/s12943-017-0688-6)
Supplement: Supplementary file 2 — A list of primers used in the reactions for real-time RT-PCR. (PDF 9 kb) [file 12943_2017_688_MOESM2_ESM.pdf]

Table S2. A list of primers used in the reactions for real-time RT-PCR.

**Real-time PCR primer:**	
TNIP1-up	CAGCAGCAGGCTAGTGTGAC
TNIP1-dn	CTGCTTGTTCACTTCCAGCA
SOCS1-up	CACATGGTTCCAGGCAAGTA
SOCS1-dn	CTACCTGAGCTCCTTCCCCT
PIAS4-up	AAGCTTCTCGTTGTTCTGTGG
PIAS4-dn	CAAGACCCTCAAGCCAGAAG
PDLIM7-up	CACGTGCCAGGTCATCTTC
PDLIM7-dn	ATGCTATGACGTGCGCTATG
TWIST1-up	TCCATTTTCTCCTTCTCTGGAA
TWIST1-dn	GTCCGCGTCCCACTAGC
MMP13-up	AACATCCAAAAACGCCAGAC
MMP13-dn	GGAAGTTCTGGCCAAAATGA
IL11-up	TGAAGACTCGGCTGTGACC
IL11-dn	CCTCACGGAAGGACTGTCTC
GAPDH-up	ATTCCACCCATGGCAAATTC
GAPDH-dn	TGGGATTTCCATTGATGACAAG
