# Supplementary material for: Oncogenic miR-210-3p promotes prostate cancer cell EMT and bone metastasis via NF-κB signaling pathway
Source: Mol Cancer. 2017 Jul 10;16:117. doi: 10.1186/s12943-017-0688-6 (PMC5504657; doi:10.1186/s12943-017-0688-6)
Supplement: Supplementary file 3 — The clinicopathological characteristics in 149 patients with prostate cancer. (PDF 54 kb) [file 12943_2017_688_MOESM3_ESM.pdf]

Table S3. The clinicopathological characteristics in 149 patients with prostate cancer

Parameters	Number of cases
Age (years)	
≤72	74
>72	75
Median	73
SD	11.5
Differentiation	
Well/moderate	69
Poor	80
Serum PSA at diagnosis, μg/mL	
<90.2	74
>90.2	75
Median	90.2
SD	384.2
Mean	303.9
Gleason grade	
≤7	78
>7	71
Operation	
TURP	20
Needle biopsy	6
TURP+PP	4
TURP+BO	30
BO	11
miR-210-3p expression	
<5.87	75
>5.87	74
Median	5.87
SD	4.73
Mean	7.49
BM-status	
BM-free	81

BM	68

Abbreviation: PSA, prostate-specific antigen; TURP, Trans Urethral Resection Prostate; PP, Prior Prostatectomy; BO, Bilateral Orchiectomies; SD, Standard deviation; IHC, Immunological Histological Chemistry; BM, Bone Metastasis.
