# Supplementary figures and images for: Oncogenic miR-210-3p promotes prostate cancer cell EMT and bone metastasis via NF-κB signaling pathway
Source: Mol Cancer. 2017 Jul 10;16:117. doi: 10.1186/s12943-017-0688-6 (PMC5504657; doi:10.1186/s12943-017-0688-6)

**Figure S1**

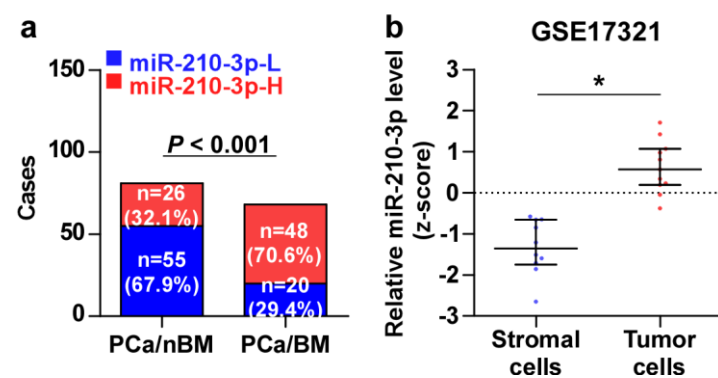

Supplement: Supplementary file 4 — miR-210-3p expression is upregulated in bone metastatic PCa tissues and cells. (a) Percentages and number of samples showed high or low miR-210-3p expression in our PCa patients with different bone metastasis. P < 0.001. (b) miR-210-3p expression was elevated in PCa cells compared with that in stromal cells in GSE17321 dataset. *P < 0.05. (PDF 62 kb) [file 12943_2017_688_MOESM4_ESM.pdf]

**Figure S2**

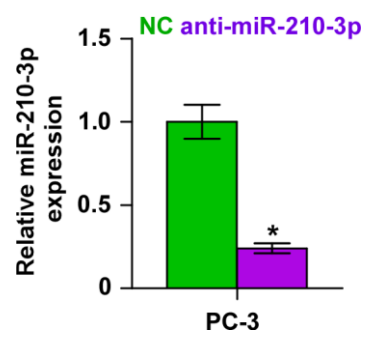

Supplement: Supplementary file 6 — Silencing miR-210-3p repressed EMT, invasion and migration in PC-3 cells in vitro. Real-time PCR analysis of miR-210-3p expression in PC-3 cells transduced with antagomiR-210-3p compared to controls. Transcript levels were normalized by U6 expression. Error bars represent the mean ± s.d. of three independent experiments. *P < 0.05. [file 12943_2017_688_MOESM6_ESM.pdf]

Figure S3

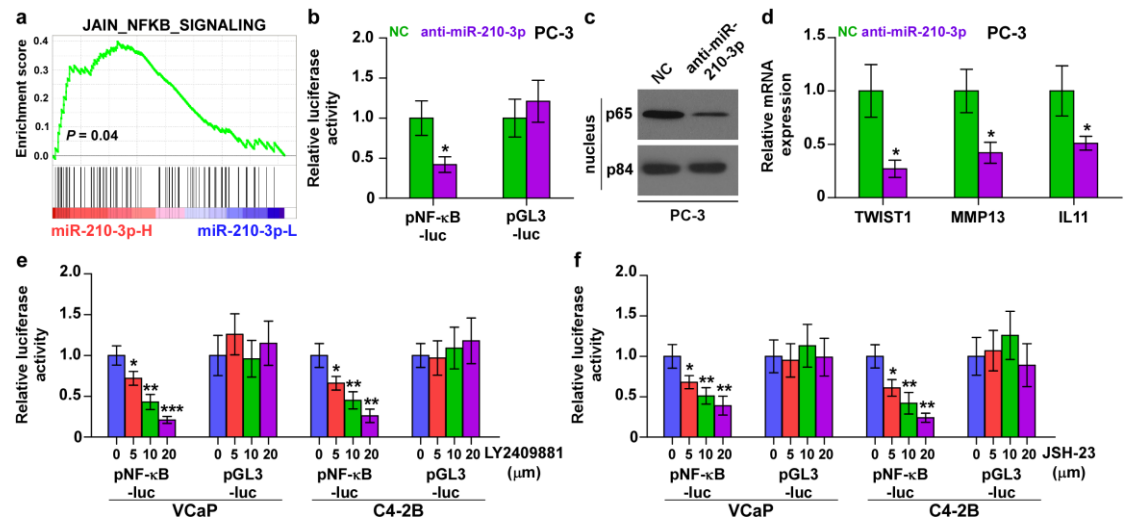

Supplement: Supplementary file 7 — Silencing miR-210-3p inhibits NF-κB signaling activity in PC-3 cells. (a) Gene set enrichment analysis (GSEA) revealed that miR-210-3p expression significantly and positively correlated with the NF-κB signaling. (b) NF-κB transcriptional activity was repressed by silencing miR-210-3p in the indicated PC-3 cells. Error bars represent the mean ± S.D. of three independent experiments. *P < 0.05. (c) Western blotting of nuclear NF-κB/p65 expression. The nuclear protein p84 was used as the nuclear protein marker. (d) Real-time PCR analysis of TWIST1, MMP13 and IL11 in the indicated cells. Error bars represent the mean ± S.D. of three independent experiments. *P < 0.05. (e and f) NF-κB signaling inhibitors LY2409881 and JSH-23 inhibited the NF-κB transcriptional activity in a dose-dependent manner in the indicated cells. Error bars represent the mean ± S.D. of three independent experiments. *P < 0.05, **P < 0.01 and ***P < 0.001. (PDF 128 kb) [file 12943_2017_688_MOESM7_ESM.pdf]

**Figure S4**

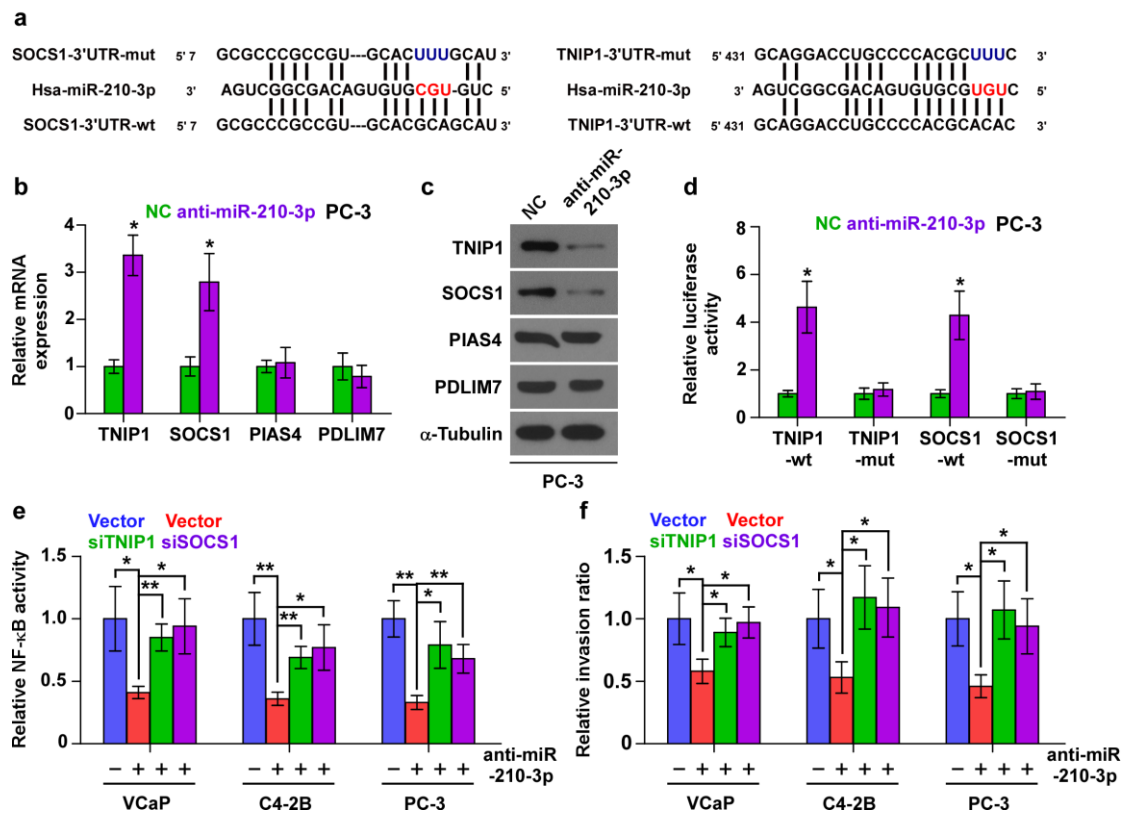

Supplement: Supplementary file 8 — miR-210-3p targets multiple negative regulators of NF-κB signaling. (a) Predicted miR-210-3p targeting sequence and mutant sequences in 3’UTR s of SOCS1 and TNIP1. (b) Real-time PCR analysis of TNIP1, SOCS1, PIAS4 and PDLIM7 expression in the indicated cells. Error bars represent the mean ± S.D. of three independent experiments. *P < 0.05. (c) Western blotting of TNIP1, SOCS1, PIAS4 and PDLIM7 expression in the indicated cells. α-Tubulin served as the loading control. (d) Luciferase assay of cells transfected with pmirGLO-3’UTR reporter of TNIP1 and SOCS1 in the miR-210-3p silencing PC-3 cells. *P < 0.05. (e and f) Individual silencing of TNIP1 and SOCS1 rescued the NF-κB activity (e) and invasion (f) abilities repressed by miR-210-3p silencing in PCa cells. *P < 0.05 and **P < 0.01. (PDF 185 kb) [file 12943_2017_688_MOESM8_ESM.pdf]

**Figure S5**

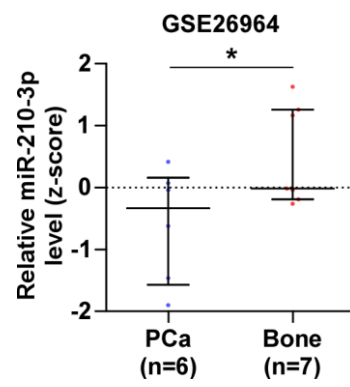

Supplement: Supplementary file 9 — miR-210-3p expression levels was markedly elevated in metastatic bone tissues compared with that in primary PCa tissues with bone metastasis (BM, n = 6; Bone, n = 7). *P < 0.05. (PDF 28 kb) [file 12943_2017_688_MOESM9_ESM.pdf]

**Figure S6**

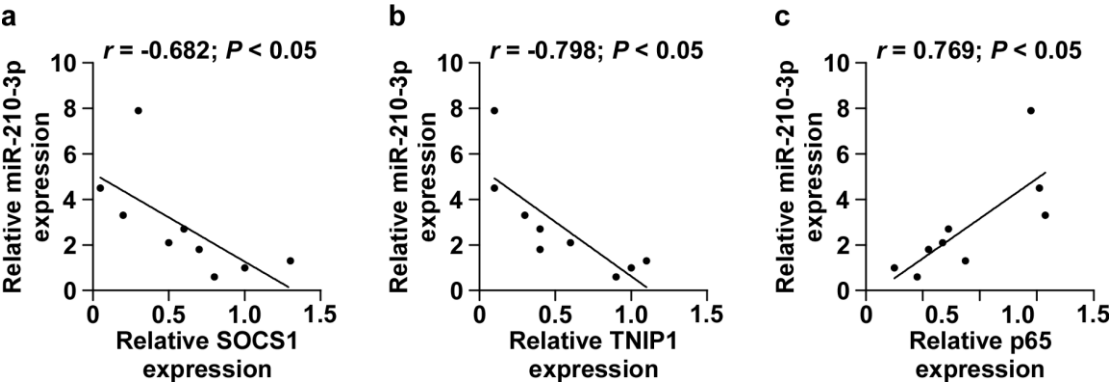

Supplement: Supplementary file 10 — Clinical correaltion of miR-210-3p with SOCS1, TNIP1 and nuclear p65 in human PCa and bone tissues. (a-c) Correlation between miR-210-3p levels and SOCS1, TNIP1 and nuclear p65 expression in PCa and bone tissues.The expression levels of SOCS1, TNIP1 and nuclear p65 were quantified by densitometry using Quantity One Software, and normalized to the levels of α-tubulin and p84, respectively. The sample 1 was used as a standard. The relative expressions of miR-210-3p and these proteins were used to perform the correlation analysis. (PDF 88 kb) [file 12943_2017_688_MOESM10_ESM.pdf]
