# Supplementary material for: Oncogenic miR-210-3p promotes prostate cancer cell EMT and bone metastasis via NF-κB signaling pathway
Source: Mol Cancer. 2017 Jul 10;16:117. doi: 10.1186/s12943-017-0688-6 (PMC5504657; doi:10.1186/s12943-017-0688-6)
Supplement: Supplementary file 5 — The relationship between miR-210-3p and clinicopathological characteristics in 149 patients with prostate cancer. (PDF 58 kb) [file 12943_2017_688_MOESM5_ESM.pdf]

Table S4. The relationship between miR-210-3p and clinicopathological characteristics in 149 patients with prostate cancer.

Parameters	Number of cases	miR-210-3p expression Low	High	*P* values
Age (years)				
≤72	74	38	36	0.805
>72	75	37	38	
Differentiation				
Well/moderate	69	34	35	0.391
Poor	80	41	39	
Serum PSA				
<90.2	75	48	27	
>90.2	74	27	47	<0.001*
Gleason grade				
≤7	78	53	25	
>7	71	22	49	<0.001*
Operation				
TURP	68	36	32	
Needle biopsy	72	35	37	0.800
TURP+PP	11	4	7	
TURP+BO	31	17	14	
BO	16	7	9	
BM-status				
nBM	81	55	26	
BM	68	20	48	<0.001*

Abbreviation: PSA, prostate-specific antigen; TURP, Trans Urethral Resection Prostate; PP, Prior Prostatectomy; BO, Bilateral Orchiectomies; SD, Standard deviation; IHC, Immunological Histological Chemistry; BM, Bone Metastasis.
